# Ultra‐Thin Hybrid Ion Exchange Interlayer for High Performance Aqueous Zn Metal Batteries

**DOI:** 10.1002/advs.202522394

**Published:** 2026-01-23

**Authors:** Tong Yang, Weijia Meng, Tan Trung Kien Huynh, Zhengyu Wang, Jiaye Ye, Yang Yang, Minh Tam Hoang, Zixuan Liu, Zijian Cai, Meiqing Guo, Jingsan Xu, Hongxia Wang

**Affiliations:** ^1^ School of Chemistry and Physics Queensland University of Technology Brisbane Queensland Australia; ^2^ Shaanxi Key Laboratory of New Transportation Energy and Automotive Energy Saving School of Energy and Electrical Engineering Chang'an University Xi'an Shaanxi China; ^3^ Institute of Applied Mechanics College of Aeronautics and Astronautics Taiyuan University of Technology Taiyuan China

**Keywords:** aqueous zn metal batteries, fluorinated carbon dots, ion‐exchange interlayer, sulfonated poly(ether ether ketone), zn anode

## Abstract

The application of aqueous zinc metal batteries (AZMBs) is limited by the poor cycling stability and undesired dendritic growth of Zn anodes. Herein, a Zn anode with an ultra‐thin sulfonated poly(ether ether ketone) (SPEEK) and fluorinated carbon dots (FCDs) hybrid interlayer is developed to enhance AZMBs performance. The SPEEK/FCD interlayer possesses excellent stability and is found to play multiple functions, including effectively regulating electrolyte solvation structure by ion‐exchange groups, promoting ion transport through the ion‐exchange channels of the hybrid SPEEK/FCD interlayer, inducing the in situ formation of a stable ZnF_2_‐rich solid electrolyte interphase (SEI), and accommodating volume fluctuations during Zn plating/stripping cycles. As a result, at 1 mA cm^−2^ and 1 mAh cm^−2^, Zn||Zn symmetric cells with the optimized SPEEK/FCD Zn electrode exhibit an ultralong lifespan of over 5500 h, much better than that of the bare Zn electrode (119 h). In addition, at 1 A g^−1^ after 1000 cycles, the Zn||V_2_O_5_ full cell with the optimized electrode retains 89.8% of its initial capacity, compared to 40.3% for the bare Zn electrode. These superior performances indicate the optimized Zn electrode is promising for next‐generation AZMBs. Also, the simple and scalable method offers a valuable reference for improving the performance of other aqueous batteries and beyond.

## Graphical abstract

An ultra‐thin SPEEK/FCD ion‐exchange interlayer modulates the Zn^2+^ solvation environment (–SO_3_^−^), provides ion‐exchange pathways that homoginized the Zn^2+^ flux, and via F‐rich chemistry forms in situ ZnF_2_ that suppresses parasitics and stabilizes the interface; its integrity relieves interfacial stress. The interlayer delivers compact Zn, long‐life symmetric and Zn||Cu cycling, and 89.8% capacity retention over 1000 full‐cell cycles.

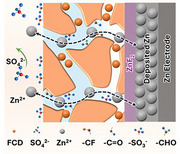

## 1 Introduction

Aqueous zinc metal batteries (AZMBs) have attracted significant attention due to their inherent safety, environmental friendliness, and the natural abundance of zinc [1, 2, 3, 4, 5]. Despite these advantages, the practical implementation of AZMBs is hindered by several critical challenges. One major issue is the uneven Zn^2+^ ion distribution at the electrode‐electrolyte interface, which promotes the formation of dendrites and compromises battery stability [6]. Additionally, parasitic side reactions, such as self‐corrosion and hydrogen evolution, accelerate anode degradation and reduce the energy efficiency of AZMBs [7, 8]. A further key limitation is the accumulation of passivation layers (e.g., Zn_4_SO_4_(OH)_6_·5H_2_O) on the Zn surface, which increases interfacial resistance and depletes active material, ultimately leading to rapid capacity fading and poor cycling reversibility [5, 9]. Addressing these issues is essential to unlocking the full potential of AZMBs for next‐generation energy storage applications.

Various strategies have been proposed to address the challenges of AZMBs, including interfacial engineering [10], electrolyte formulations [11, 12, 13], electrode structure modification [14, 15, 16], and selective separators [17]. Among such strategies, tailoring the Zn/electrolyte interface is particularly effective in enhancing Zn anode performance and boosting the cyclability of AZMBs, as it allows precise control over ion transport, solvent interactions, and the local electric field—key factors for achieving uniform and reversible Zn deposition [18, 19]. Building on this rationale, recent studies demonstrate that well‐designed interlayers can precisely steer Zn^2+^ flux/desolvation and homogenize the local electric field to realize uniform, reversible Zn deposition. Representative approaches include inorganic solid‐electrolyte coatings such as Na super‐ionic conductor‐type NaTi_2_(PO_4_)_3_ (NTP), which acts as an “ion‐passable fence;” a ∼20–25 µm NTP layer refines Zn nucleation and enables nearly 100% CE over 10 000 cycles in Zn‐MnO_2_ full cells [20]. Ultrafine artificial interphases made by atomic layer deposition (ALD), e.g., ∼10 nm In_2_O_3_ with a wide bandgap that blocks electron leakage and raises Zn adsorption energy, suppress hydrogen evolution reaction (HER)/corrosion and deliver 2800 h in symmetric cells with over 99.7% CE (Coulombic efficiency) in Zn||Cu [21]. Fluoride‐containing interlayers, exemplified by MgF_2_ nanocrystals on monolayer MoS_2_, evolve in situ to a ZnF_2_‐rich interphase that homogenizes Zn^2+^ flux, lowers polarization, and stabilizes both symmetric and V_2_O_5_ full cells [22]. Alloy/conversion skins, such as a porous ZnP matrix, accelerate Zn^2+^ desolvation/transport and promote homogeneous nucleation [23]. Zincophilic carbon scaffolds, e.g., freestanding N, O‐doped carbon‐nanofiber membranes, capture Zn ions, reduce nucleation overpotential, and sustain long cycling at high current densities while boosting full‐cell performance [24]. Finally, organic‐inorganic hybrids merge ion selectivity with mechanical robustness; a Nafion/boehmite layer with 2.7 Å galleries and hydroxyl‐driven zincophilicity achieves 9000 cycles at 5 mA cm^−2^, and even operates stably in seawater‐based electrolytes [25]. Collectively, these advances substantiate that synchronizing ion‐flux regulation, desolvation control, and local‐field homogenization at an electron‐blocking interface is decisive for durable Zn anodes, setting the stage for the following survey of interlayers and surface modifications.

In this context, various materials have been employed as inerlayers or for surface modification, including polymeric, inorganic, and scaffold‐based architectures, among others. These strategies primarily function by modifying the solvation structure, facilitating ion transport, stabilizing the solid electrolyte interphase (SEI), and regulating Zn plating/stripping behaviour. However, most reported interlayers exhibit limited functionality and fail to address the multifaceted challenges of Zn anodes, such as poor Zn^2+^ selectivity, mechanical instability, and insufficient interfacial regulation. While effective in specific aspects, monofunctional interlayers are limited and do not provide a comprehensive remedy for Zn anode instability. Accordingly, the interfacial layer should couple Zn‐nucleation control, ion‐transport/solvation optimization, SEI stabilization, and stress buffering in a thin, robust, ion‐selective, and scalable architecture, thereby enabling dendrite‐free, durable Zn anodes for high‐performance AZMBs.

**SCHEME 1:**
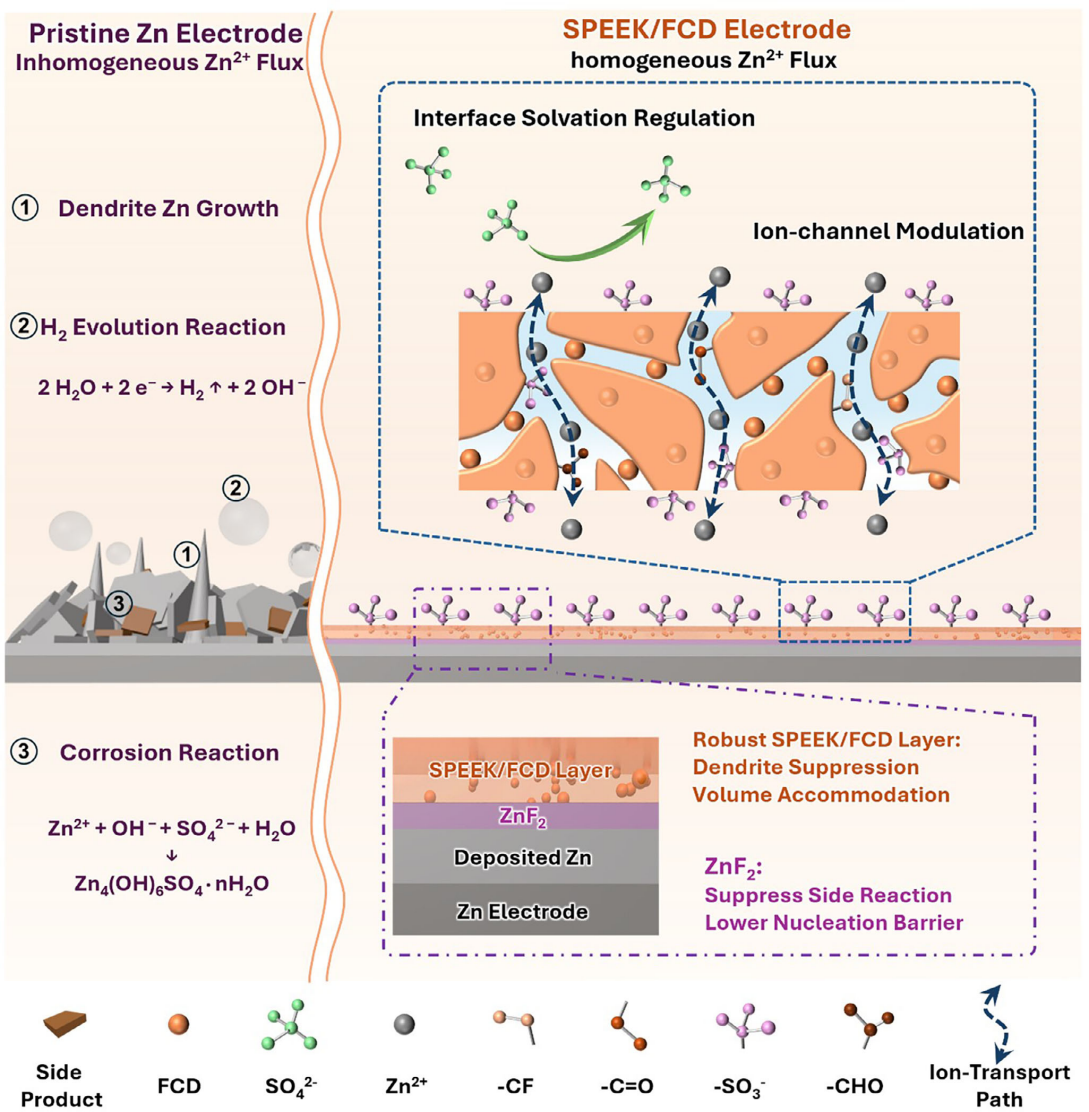
Schematic illustration of the main functions of tailored SPEEK/FCD interlayer on the surface of zinc metal anode in improving AZMBs electrochemical performance.

Herein, an ultra‐thin sulfonated poly(ether ether ketone)/fluorinated carbon dots (SPEEK/FCD) hybrid ion‐exchange interlayer is tailored to the zinc metal surface to enhance its interfacial properties, including homogenized Zn^2+^ flux, reduced nucleation overpotential, and uniform, compact zinc, thereby boosting the performance of corresponding AZMBs. Scheme 1 shows the multifunctional roles of the SPEEK/FCD interlayer in AZMBs. First, the enriched sulfonic acid groups (ion‐exchange groups) in SPEEK regulate the electrode/electrolyte interface by modifying the solvation structure of Zn^2+^, thereby enhancing its transport and deposition kinetics. Second, FCDs act as additives that refine the SPEEK‐drived ion‐exchange domains size and continuity, enhancing cation selectivity and yielding a steadier Zn^2+^ flux that homogenizes the interfacial field and enables uniform, compact Zn deposition. Third, the fluorine‐rich microenvironment induced by FCDs promotes the in situ formation of a ZnF_2_‐based SEI. This inorganic interface not only suppresses parasitic reactions but also stabilizes the interfacial energy distribution, reducing the local nucleation barrier and thereby lowering the nucleation overpotential. Finally, the hybrid ion‐exchange interlayer exhibits excellent mechanical integrity, which alleviates stress accumulation during Zn plating/stripping and prevents localized strain and instability, thus effectively suppressing Zn dendrite formation. Under the synergistic effects, the Zn anode with the SPEEK/FCD interlayer possesses excellent suppression of side reactions and zinc dendrites, which is supported by COMSOL simulations. Compared with the bare Zn anode, the AZMBs with the optimized SPEEK/FCD Zn anode exhibit significantly improved cycling stability and rate performance.

## 2 Result and Discussion

### 2.1 Interfacial Modification Toward Improved Wettability and Stability

The Zn electrodes were modified using either pristine SPEEK or SPEEK mixed with additives (carbon dots, denoted as CDs, or fluorinated CDs, denoted as FCDs). These modified electrodes are denoted as *x*SP*y*(F)CD, where *x* and *y* represent the weight percentages of SPEEK and (F)CDs relative to the dispersion, respectively. Based on optimization, the composition with 4% SPEEK and 2% additive was selected as the optimal formulation based on systematic optimization. The detailed optimization and preparation procedure is provided in the (Figures S4 and S5). Based on the optimized formulation, the modified Zn electrodes were classified into three representative types: 4SP, 4SP2CD, and 4SP2FCD. In the following sections, their electrochemical and physicochemical properties are systematically investigated to determine the most effective configuration.

**FIGURE 1:**
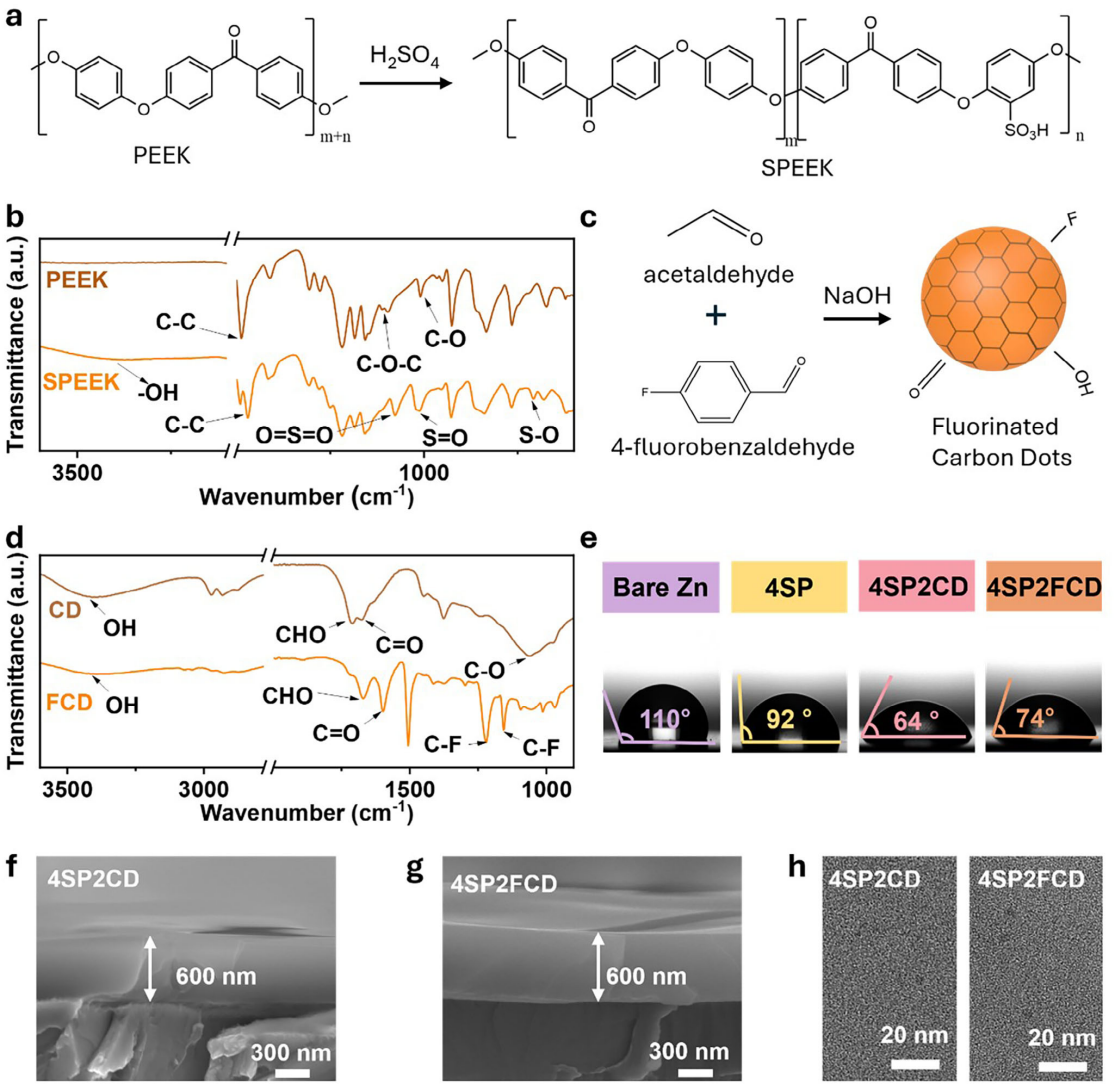
Multifunctional interface characterization. (a) The synthesis reaction of SPEEK. (b) FTIR spectra of PEEK and SPEEK. (c) The synthesis reaction of FCDs. (d) FTIR spectra of CDs and FCDs. (e) Contact angles of 2 mol L^−1^ ZnSO_4_ on bare Zn, 4SP, 4SP2CD, and 4SP2FCD electrodes. Cross‐sectional SEM image of a 4SP2CD electrode (f) and 4SP2FCD electrode (g) with a ∼600 nm thick interlayer. (h) TEM image of the 4SP2CD (left) and 4SP2FCD (right) coating.

SPEEK was synthesized by the sulfonation of poly(ether ether ketone) with the introduction of −SO_3_H groups (ion‐exchange groups) (Figure 1a) [26]. The −SO_3_H group is a cation‐exchange group and facilitates the selective transport of zinc ions. The successful synthesis of SPEEK is confirmed by Fourier transform infrared spectroscopy (FTIR) analysis. The FTIR spectrum (Figure 1b) shows multiple characteristic peaks of SPEEK: the O─H stretching vibration located at 3436 cm^−1^, the aromatic C─C stretching at 1474 cm^−1^, the O═S═O symmetric stretching at 1074 cm^−1^, the S═O stretching at 1017 cm^−1^, and the S─O stretching at 706 cm^−1^ [27]. Meanwhile, FCDs were synthesized by an aldol condensation reaction of acetaldehyde and 4‐fluorobenzaldehyde (Figure 1c). The FTIR spectrums exhibit distinct C─F stretching peaks at 1155 and 1220 cm^−1^, which distinguished them from non‐fluorinated CDs as shown in Figure 1d [28]. The surface of Zn foil was deposited with SPEEK/FCD interlayer by spin‐coating. The chemical structure of 4SP2CD and 4SP2FCD interlayers were also analyzed (Figure S2). For the 4SP2CD interlayer, the characteristic peak of O═S═O symmetric stretching vibration in −SO_3_H of SPEEK appears at 1078.6cm^−1^, while the −CHO functional group from CDs is observed around 1714 cm^−1^. In the case of the 4SP2FCD interlayer, in addition to the characteristic peaks for O═S═O and −CHO, a distinct C─F stretching peak further confirms the successful incorporation of FCDs in the SPEEK polymer matrix. These FTIR results indicate that SPEEK/CDs and SPEEK/FCD were successfully spin‐coated onto the surface of Zn foil.

The electrolyte interfacial wettability of various electrodes was evaluated using contact angle measurements in the presence of a 2 mol L^−1^ ZnSO_4_ solution. As shown in Figure 1e, the bare Zn surface exhibits poor wettability, with a contact angle of 110°, indicating weak electrolyte affinity. Upon the surface of Zn with a 4SP (pure SPEEK) interlayer, the wettability was improved slightly, with the contact angle reducing to 92°. This is mainly attributed to the hydrophilic −SO_3_H groups [29]. With further incorporation of CDs (4SP2CD), the contact angle decreases to 64°, indicating a significantly enhanced wettability. This is primarily due to the hydroxyl (−OH) and carboxyl (−COOH) functional groups in CDs [30]. However, replacing CDs with FCDs (4SP2FCD) leads to a slight increase in the contact angle to 74°, which can be attributed to the lower surface energy imparted by the hydrophobic C−F bonds in FCDs [31]. Although the contact angle slightly increases compared to CDs, the 4SP2FCD electrode still exhibits markedly improved wettability over bare Zn and 4SP electrodes, enabling more uniform electrolyte spreading, enhanced Zn^2+^ distribution, and reduced interfacial resistance. Cross‐sectional scanning electron microscopy (SEM) images reveal a distinct difference between the electrodes. As shown in Figure S6, the thickness of the interlayer (0–2 µm) can be well manipulated by controlling the content of SPEEK in solution (0–10 wt.%). The interlayer thickness of the 4SP2CD (Figure 1f) and 4SP2FCD (Figure 1g) electrodes containing 4 wt.% SPEEK was approximately 600 nm. Transmission electron microscopy (TEM) analysis further confirms the size of the CDs and FCDs (Figure S3a,b) and uniform distribution of CDs and FCDs with an average diameter of 5 nm within the SPEEK matrix (Figure 1h).

To assess the long‐term stability of the interlayer, bare Zn, 4SP, 4SP2CD, and 4SP2FCD electrodes were immersed in a 2 mol L^−1^ ZnSO_4_ solution for extended durations. After 7 days, the bare Zn surface exhibited a significant accumulation of loose by‐products (Zn_4_SO_4_(OH)_6_·5H_2_O) (Figure S7b), whereas 4SP, 4SP2CD, and 4SP2FCD electrodes (Figure S7c–e) remained smooth and unchanged from their initial state (Figure S7a), with a thin protective interlayer still visibly covering the Zn surface. Furthermore, after an extensive stability test over 30 days, the 4SP2FCD electrode surface remained well‐preserved (Figure S8c), while the bare Zn electrode developed by‐product deposits reaching 100 µm in diameter (Figure S8a). The morphological observations are further corroborated by X‐ray diffraction (XRD) analysis (Figure S8b,d), which confirms the formation of a Zn_4_SO_4_(OH)_6_·5H_2_O by‐product on bare Zn that would increase the interfacial resistance and decrease the cycling stability of the electrode, whereas the surface of the 4SP2FCD electrode maintained its structural integrity with only a small amount of by‐product formation.

### 2.2 Solvation Regulation and Ion‐Exchange Interlayer

**FIGURE 2:**
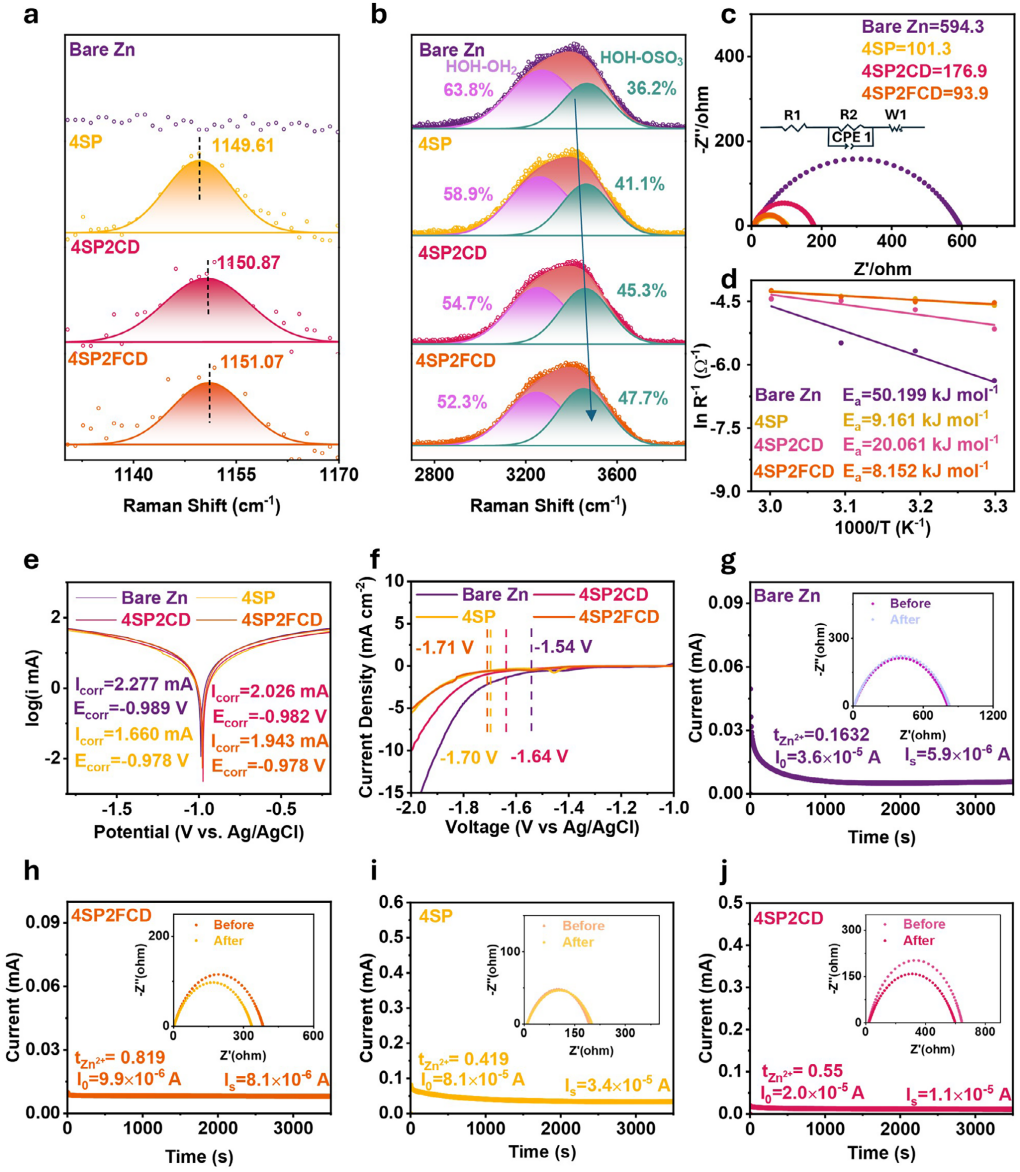
Solvation structure modification and anticorrosion effect. (a,b) Raman spectra showing shifts in SO_3_^−^, and O─H vibration bands. (c) EIS spectra of Zn||Zn symmetric cells with different electrolytes at 30 °C. (d) Activation energies (*E*_a_) for Zn^2+^ deposition, derived from *R*_ct_ at varying temperatures. (e,f) LSV curves assess corrosion resistance and hydrogen evolution. (g–j) Chronoamperometry (CA) curves and Zn^2+^ ion transfer numbers. Insets in (g, h, i, j) are the EIS spectra of the cells before and after the CA test.

Raman spectroscopy was employed to investigate the solvation structures of a 2 mol L^−1^ ZnSO_4_ electrolyte on both bare and modified Zn electrodes. As shown in Figure S9, the SO_4_^2−^ vibration band shifts from 982.6 cm^−1^ to approximately 982.0 cm**^−^** ^1^ in 4SP, 4SP2CD, and 4SP2FCD electrodes, indicating that the molecular interactions between Zn^2+^ and SO_4_^2−^ are disrupted in the presence of the SPEEK‐based interface. As shown in Figure 2a, the SO_3_^−^ vibration band shifts from 1149.6 cm**^−^** ^1^ to 1150.8 and 1151.1 cm**^−^** ^1^ in 4SP2CD and 4SP2FCD interlayers, respectively, indicating that Zn^2+^ polarization modulates S─O dipole interactions within the interlayers [29]. Notably, this blueshift suggests strengthened interactions between Zn^2+^ species and the sulfonate groups, consistent with cation‐induced polarization effects commonly observed in sulfonated polymer systems reported in the literature. With the incorporation of CDs and FCDs, additional surface functional groups further enhance Zn^2+–^SO_3_^−^ electrostatic interactions, restricting the vibrational freedom of the S–O bonds and resulting in the observed progressive blueshift, while concurrently reducing the relative contribution of Zn^2+–^H_2_O coordination within the interlayer region [29, 32, 33]. Furthermore, Raman peaks associated with O─H vibrations, particularly the HOH−OH_2_ stretching mode at 3270 cm**^−^** ^1^ and the HOH−OSO_3_^2−^ dominated band at 3460 cm**^−^** ^1^, shift to higher frequencies in the 4SP, 4SP2CD, and 4SP2FCD electrodes (Figure 2b). This shift indicates a decrease in the proportion of free water molecules, highlighting the effectiveness of the SPEEK‐based interlayer in modulating solvation structures [34]. To further validate this observation, zeta potential measurements were conducted to assess the surface charge properties of CDs and FCDs in water (Figure S10). The results show that FCDs exhibit a significantly lower zeta potential (−12.1 mV) compared to CDs (−3.62 mV), indicating a stronger electrostatic interaction with Zn^2+^, which promotes ion adsorption and transport at the anode interface.

To further evaluate the impact of the SPEEK‐based interlayer on Zn^2+^ ion deposition kinetics, the activation energies (*E*_a_) of various electrodes were calculated using the Arrhenius equation. This analysis involved fitting the electrochemical impedance spectra, specifically the charge transfer resistance (*R*_ct_), of different Zn electrodes in Zn||Zn symmetric cells measured under different temperatures (Figure 2c). As shown in Figure 2c, 4SP2CD exhibits the highest interfacial impedance, followed by 4SP, while 4SP2FCD shows a slightly lower impedance than 4SP. The increased impedance of 4SP2CD can be attributed to the introduction of carbon dots refines the SPEEK‐driven ion‐exchange domains by tuning domain size and continuity, thereby increasing interfacial resistance. In contrast, the lower impedance of 4SP2FCD can be attributed to the formation of a zincophilic ZnF_2_ interphase during initial cycling, which facilitates interfacial charge transfer and compensates for the impedance increase associated with domain refinement. The calculation details of *E*_a_ are provided in the Supporting Information. As shown in Figure 2d, the *E*_a_ of the bare Zn electrode is 50.19 kJ mol^−1^, indicating sluggish charge transfer kinetics. In contrast, the 4SP2FCD electrode exhibits a significantly reduced activation energy of 8.15 kJ mol^−1^, highlighting its ability to facilitate Zn^2+^ deposition. The activation energies for 4SP (9.16 kJ mol^−1^) and 4SP2CD (20.06 kJ mol^−1^) were also determined, revealing that 4SP2CD exhibits a slightly higher *E*_a_ and *R*_ct_ compared to 4SP. This can be attributed to CDs, which partially obstruct ion channels within the SPEEK matrix, limiting Zn^2+^ transport efficiency. However, in 4SP2FCD this potential drawback associated with confined domains is largely suppressed, as the low FCD loading (2 wt.%) minimizes domain‐induced transport perturbations, while the fluorine‐rich surface chemistry promotes the in situ formation of a ZnF_2_‐rich SEI layer that governs interfacial Zn^2+^ transport. This protective interphase significantly lowers the desolvation barrier and stabilizes Zn^2+^ flux at the interface, thereby restoring ion accessibility and enhancing Zn^2+^ transport through the interlayer [35]. The substantial decrease in activation energy indicates the effectiveness of the 4SP2FCD interlayer in modulating interfacial ion dynamics and accelerating Zn^2+^ transport.

The anticorrosion behaviour of different electrodes was evaluated using electrochemical linear sweep voltammetry (LSV). All modified electrodes exhibited lower corrosion currents and more positive corrosion potentials compared to bare Zn (Figure 2e). The bare Zn electrode displayed a corrosion current (*I*_corr_) of 2.277 mA and a corrosion potential (*E*_corr_) of −0.989 V. The 4SP electrode showed a reduced *I*_corr_ of 1.660 mA and a slightly more positive *E*_corr_ (−0.978 V), indicating improved interfacial protection by the SPEEK layer. The 4SP2CD electrode exhibits a moderately increased *I*_corr_ of 2.026 mA under the *E*_corr_ of −0.982 V, suggesting inferior protection. Although the CDs are uniformly dispersed, the relatively high loading may introduce electrochemically active sites or micro‐galvanic junctions, which facilitate localized corrosion. In contrast, the 4SP2FCD electrode achieveed the lower *I*_corr_ (1.943 mA) and a more positive *E*_corr_ (−0.978 V), indicating superior anticorrosion performance by balancing barrier densification and minimizing these adverse electrochemical effects.

The hydrogen evolution behaviour of the electrodes was examined to assess their interfacial stability. As shown in Figure 2f, the HER potential of the 4SP2FCD (−1.71 V) electrode is less than that of bare Zn (−1.54 V), 4SP (−1.70 V), and 4SP2CD (−1.64 V) electrodes. The more negative HER potential indicateed that 4SP2FCD electrode possessed a wider electrochemical stability window, making it less prone to hydrogen evolution. In contrast, bare Zn exhibited the most positive HER onset, implying a higher tendency for parasitic reactions at the surface. Moreover, the ionic conductivity of 4SP2FCD interlayer was comparable to that of 4SP and 4SP2CD interlayer, indicating a negligible effect of the small amounts of FCDs on ion transport (Figure S11).

Chronoamperometry (CA) tests were employed to study the Zn deposition behaviour in Zn||Zn symmetric cells. The cell with the 4SP2FCD electrode, under a constant overpotential of 20 mV for 3600 s, exhibited a Zn^2+^ ion transfer number of 0.819, which is significantly higher than that of the bare Zn electrode (0.163) in a 2 mol L^−1^ ZnSO_4_ electrolyte (Figure 2g,h). The enhancement was also observed in the CA plots of 4SP (*t*_Zn_^2+^ = 0.419, Figure 2i) and 4SP2CD (*t*_Zn_^2+^ = 0.55, Figure 2j) electrodes. Although the steady‐state current decreases from 4SP to 4SP2CD and 4SP2FCD, indicating refined and more confined SPEEK‐derived ion‐exchange domains, a pronounced enhancement in Zn^2+^ transference is still observed upon additive incorporation. The enhancement can be attributed to solvation structure modifications and the presence of ion transport channels within the SPEEK/FCD interlayer. Specifically, the −SO_3_^−^ groups in SPEEK promote selective transport of Zn^2+^, while weakening its interaction with SO_4_^2^ **^−^**.

Taken together, the above results verify that the SPEEK matrix operates as an ion‐exchange interlayer. Fixed sulfonate (−SO_3_^−^) sites create cation‐selective domains that perturb the Zn^2+^ solvation structure and assist interfacial desolvation, as reflected by the SO_4_^2−^ band blueshift, the SO_3_^−^ vibration shift, and the increased HOH–OSO_3_^−^ contribution in the O─H region. Consistently, EIS and Arrhenius analyses show lowered *R*_ct_ and a markedly reduced activation energy for Zn deposition, while LSV/HER and CA measurements indicate mitigated corrosion/HER and smoother plating transients. Overall, this ion‐exchange environment homogenizes interfacial Zn^2+^ flux and stabilizes Zn deposition [36].

### 2.3 Nucleation Enhancement and In Situ ZnF_2_ SEI Formation

**FIGURE 3:**
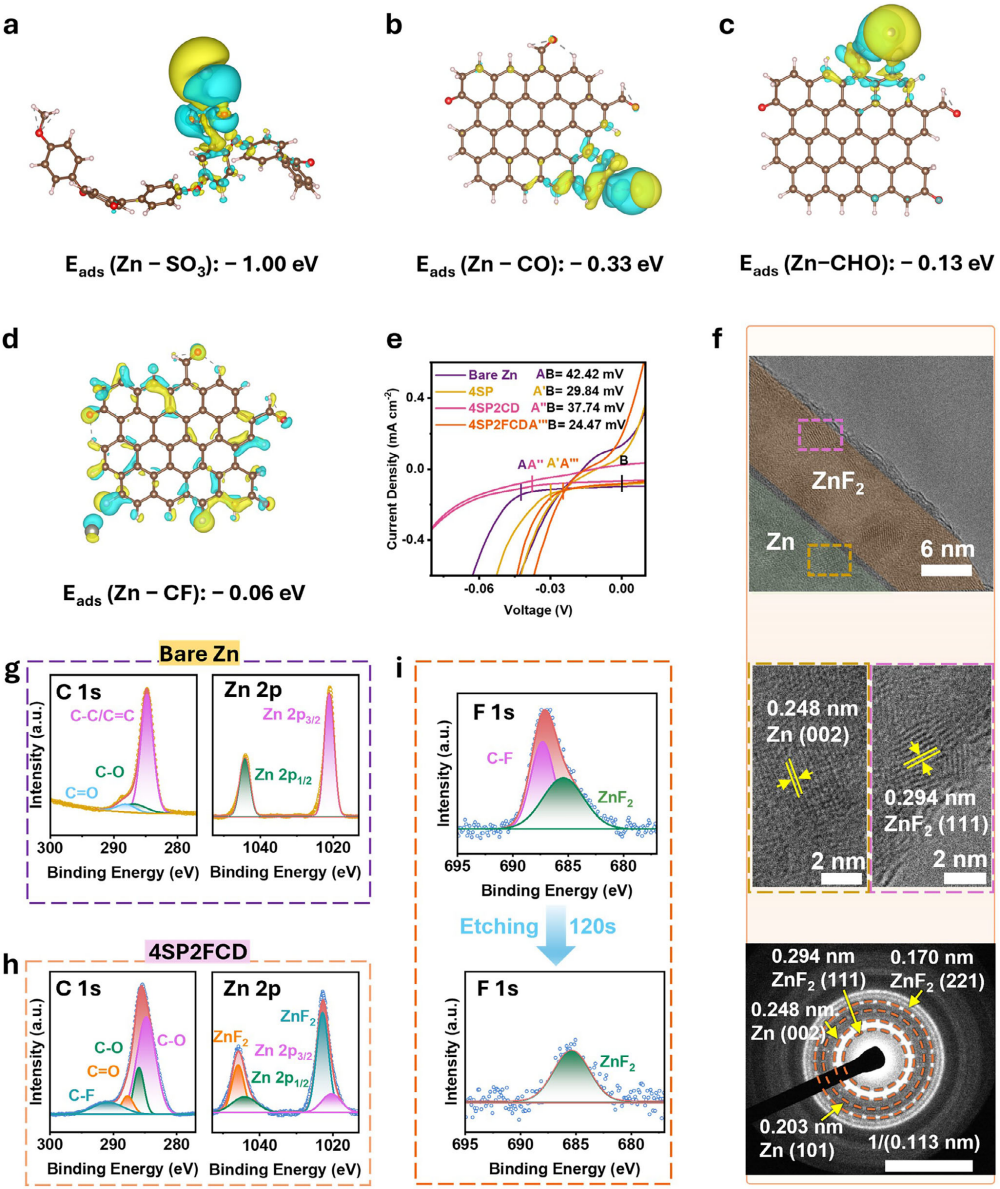
Zincphilic channel and nucleation assistance. DFT‐calculated adsorption energies (E_ads_) of Zn^2+^ on representative functional groups: (a) −CHO (−0.13 eV), (b) −CO (−0.33 eV), (c) −SO_3_H (−1.00 eV), and (d) −CF (−0.06 eV). (e) Nucleation overpotential (NOP) from CV tests for bare Zn and modified electrodes. (f) TEM image and SAED diffraction pattern of the 4SP2FCD electrode after 5 cycles under 0.2 mA cm^−2^, 0.2 mAh cm^−2^. HR‐TEM image of Zn deposits on 4SP2FCD, showing lattice fringes. (g,h) XPS spectra of bare Zn and 4SP2FCD after cycling, highlighting C─F and ZnF_2_ signals. (i) XPS depth profiling after etching, showing changes in fluorine species at the interface.

Density Functional Theory (DFT) calculations were conducted to evaluate the adsorption energies of Zn^2+^ on different functional groups present in the SPEEK‐based interlayer, including −SO_3_H (SPEEK), −CHO, −CO (CDs), and −C─F (FCDs). The adsorption energy of Zn^2+^ on a bare Zn surface is −1.69 eV (Figure S12), indicating strong Zn^2+^ affinity, which could lead to uncontrolled Zn nucleation and uneven deposition. Contrastingly, the functional groups in SPEEK, CDs, and FCDs possess relatively lower adsorption energies for Zn^2+^ (Figure 3a–d), such as approximately −1.00 eV (−SO_3_H group in SPEEK), −0.33 eV (−CO group in CDs), −0.13 eV (−CHO group in CDs or FCDs), and −0.06 eV (−C─F group in FCDs). These indicate that, besides inducing uniform transport of zinc ions, the functional groups in the ion‐exchange interfacial layer do not anchor the zinc ions nor impede their transport. Instead of undergoing rapid and uncontrolled nucleation on bare Zn, Zn^2+^ transport becomes homogeneous within the SPEEK‐based interlayer, where sulfonic acid groups define cation‐selective ion‐exchange pathways by providing continuous negatively charged domains that preferentially coordinate and guide Zn^2+^ migration, as widely reported for sulfonated polymers. Meanwhile, the incorporation of FCD additives further refines these pathways by tuning domain size and continuity, thereby enhancing overall cation selectivity [37, 38]. The corresponding electrostatic potential maps (Figure S13) further support this distribution, showing clear differences in surface polarity of CDs, FCDs, SPEEK, and deprotonated SPEEK [39, 40].

The nucleation overpotential (NOP) of Zn deposition on different electrodes was analysed using cyclic voltammetry (CV) tests. As shown in Figure 3e, the 4SP2FCD electrode exhibited a minimum NOP (24.47 mV) among these electrodes, indicating a lower energy barrier for Zn nucleation and a more favourable nucleation environment. In comparison with the NOP of 4SP and 4SP2CD electrodes, the 4SP electrode (29.84 mV) exhibited a moderate reduction in NOP, which can be attributed to the sulfonic acid (−SO_3_H) groups in SPEEK attracting Zn^2+^ ions through electrostatic interactions and coordination. In addition, the negatively charged SPEEK matrix forms ion‐selective channels that preferentially allow divalent Zn^2+^ to pass while repelling anions such as SO_4_^2−^, thereby concentrating Zn^2+^ at the interface and promoting nucleation. However, after incorporating CDs, the NOP unexpectedly increased to 37.74 mV, likely due to CDs partially obstructing Zn^2+^ transport channels in the SPEEK layer, thereby limiting ion accessibility and increasing the nucleation barrier. Further investigations into the effect of CDs content on NOP revealed that 4SP4CD exhibited an even higher NOP of 59.58 mV, confirming the hindrance of CDs to Zn nucleation (Figure S14).

The significant reduction in NOP for 4SP2FCD can be attributed not only to the optimized Zn^2+^ adsorption energy but also to the formation of a zincophilic SEI layer, which plays a crucial role in lowering nucleation barriers. Previous studies have demonstrated that ZnF_2_‐rich SEI layers effectively reduce NOP by stabilizing Zn nucleation [41]. In this work, the fluorinated functional groups (−C─F) in FCDs promote the in situ formation of ZnF_2_, further improving nucleation kinetics and promoting uniform Zn growth. The TEM and selected area electron diffraction (SAED) were employed to investigate the morphological and structural features of the ZnF_2_‐rich interphase and Zn deposition. For the 4SP2FCD electrode, Zn and ZnF_2_ nanocrystals are distributed across the interface (top of Figure 3f), with ZnF_2_ more densely concentrated near the surface region, suggesting the in situ formation of a ZnF_2_‐rich interphase layer. High‐resolution TEM (HR‐TEM) images (middle of Figure 3f) reveal distinct lattice fringes with spacings of 0.248 and 0.294 nm, corresponding to the Zn (002) and ZnF_2_ (111) planes, respectively. The corresponding SAED pattern (bottom of Figure 3f) further confirms the crystalline structure, showing diffraction rings indexed to Zn (002) at 0.248 nm, Zn (101) at 0.203 nm, as well as ZnF_2_ (111) at 0.294 nm and ZnF_2_ (221) at 0.170 nm. These results demonstrate the formation of a surface‐enriched ZnF_2_ interphase and the simultaneous growth of oriented Zn deposits beneath, which facilitate stable Zn nucleation and uniform deposition.

X‐ray photoelectron spectroscopy (XPS) analysis was conducted on bare Zn and 4SP2FCD electrodes after five cycles. The XPS spectra of the bare Zn electrode (Figure 3g) show characteristic Zn and C peaks within their expected binding energy regions. In contrast, the 4SP2FCD electrode (Figure 3h) displays a distinct −C─F spectral region, indicating the presence of fluorinated carbon species. The deconvolution of this peak reveals contributions from both covalent C─F bonds and semi‐ionic −C─F bonds from the FCDs [42, 43]. The latter are relatively weaker and more prone to dissociation during cycling. Furthermore, the Zn 2p region exhibits a clear ZnF_2_ peak that agrees with the TEM images, reconfirming the in situ formation of ZnF_2_ at the interface. The depth profiling of XPS using argon etching was subsequently conducted on the 4SP2FCD electrode to examine the spatial distribution of fluorinated species. After argon etching for 120s, the disappearance of −C─F signal suggests removal of the 4SP2FCD layer, leaving behind a dominant ZnF_2_ signal in the F 1s spectrum (Figure 3i). This transformation confirms that after five cycles of plating/stripping, the semi‐ionic C─F bonds gradually dissociate and release F^−^ ions, which react with Zn^2+^ to form a stable ZnF_2_‐rich interphase [42]. Importantly, Zn deposition does not occur within or on top of the 4SP2FCD interlayer; instead, a ZnF_2_‐rich SEI is formed beneath the polymer film, and Zn is subsequently deposited underneath this interphase on the metallic Zn substrate. These results demonstrate that the fluorine species from FCDs contribute to the formation of a protective ZnF_2_ layer, which acts as a Zn^2+^‐selective and kinetically favorable interphase [44, 45, 46], thereby playing a crucial role in stabilizing Zn deposition, lowering NOP, and mitigating side reactions.

### 2.4 Volume Accommodation and Deposition Behavior

Following comprehensive structural, interfacial, and electrochemical evaluations, 4SP2FCD was identified as the most promising candidate. We now investigate the 4SP2FCD electrode independently to gain deeper insights, with bare Zn serving as a reference.

Volume accommodation of the SPEEK/FCD interlayer plays a critical role in stabilizing Zn metal anodes by mitigating the stress induced by repeated Zn plating/stripping cycles. Without sufficient mechanical flexibility, interfacial stress accumulation can lead to cracking, delamination, and uneven Zn deposition, ultimately accelerating capacity decay. Tensile tests (Figure S15) show that the SPEEK/FCD interlayer exhibits a higher tensile strength (334 kPa) and greater deformation tolerance than that of pure SPEEK (200 kPa), indicating enhanced mechanical robustness for better accommodation of Zn volume changes during cycling.

**FIGURE 4:**
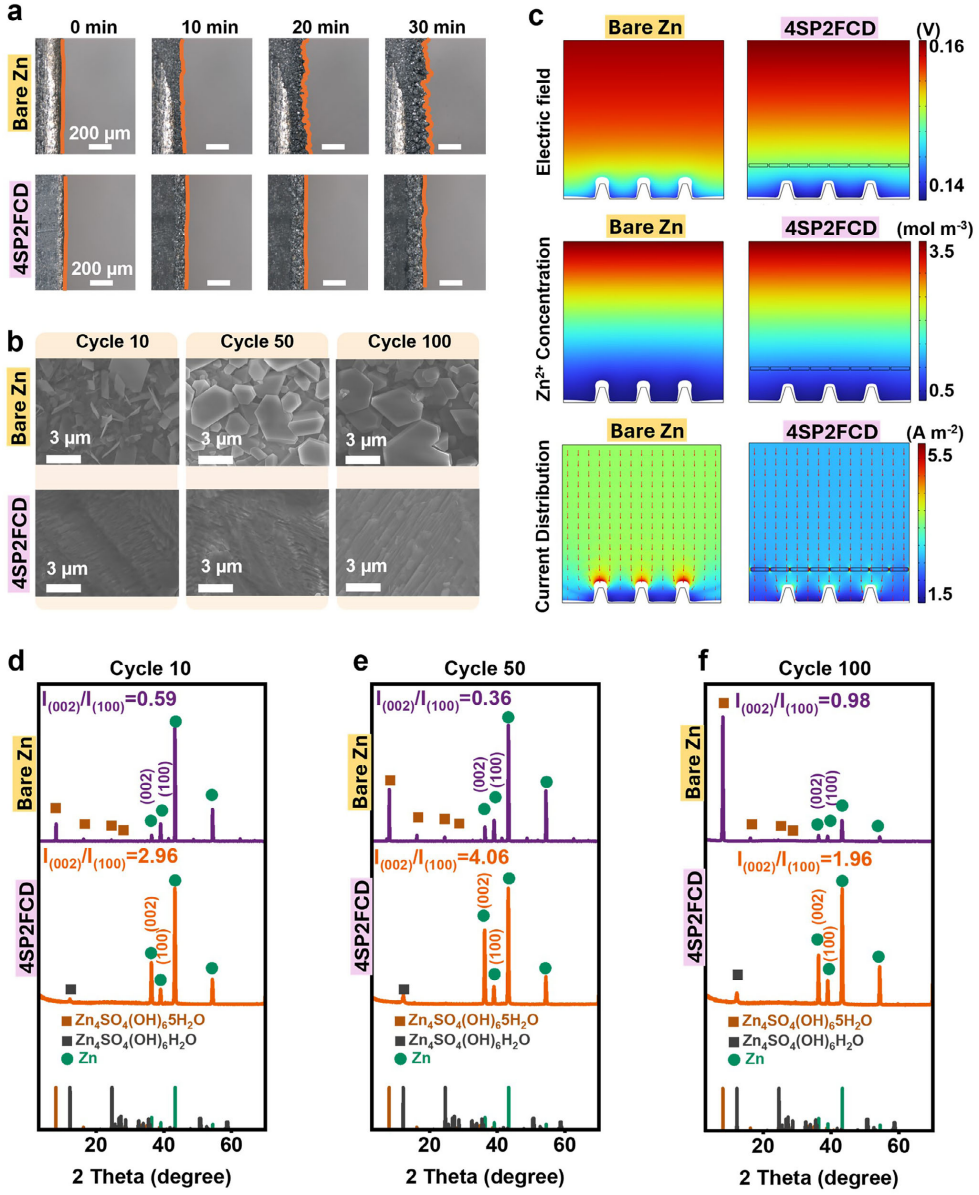
(a) In situ optical microscopy images of Zn deposition on bare Zn and 4SP2FCD electrodes over 30 min of plating. (b) SEM images of Zn electrodes after 10, 50, and 100 cycles, comparing surface morphology between bare Zn and 4SP2FCD electrode. (c) COMSOL simulation results comparing the bare Zn and 4SP2FCD electrode after 1000 s: electric field distribution, Zn^2+^ ion concentration, and current density distribution. XRD patterns of Zn electrodes after (d) 10, (e) 50, and (f) 100 cycles at 1 mA cm^−2^.

An in situ optical microscopy system was employed to monitor the morphological changes of the Zn anode during continuous plating for 30 min. As shown in Figure 4a, on the bare Zn electrode surface, non‐uniform Zn nucleation occurred within the first 10 min, accompanied by the formation of small irregular features. Over time, significant surface fluctuations appeared, and after 30 min, large protrusions and dendrites were clearly visible, indicating uncontrolled Zn deposition and severe volume fluctuations. In contrast, the 4SP2FCD electrode exhibited a more uniform and stable deposition process, with no noticeable dendritic features or irregular growth throughout the plating period. These results confirmed that the SPEEK/FCD interlayer effectively regulates Zn deposition and possesses good mechanical stability to accommodate volume expansion, thus inhibiting the uncontrolled growth of zinc dendrites.

The superior zinc deposition stability of the 4SP2FCD electrode was further confirmed through SEM analysis after 10, 50, and 100 cycles. The bare Zn electrode developed a progressively rougher morphology over cycling, transitioning from nanoflake‐covered surfaces at 10 cycles to large and compact hexagonal bulks at 100 cycles, indicating severe side reactions and uneven Zn accumulation (the top row of Figure 4b). In contrast, the Zn deposits on the 4SP2FCD electrode remain dense and smooth across all cycles, suggesting that the SPEEK/FCD interlayer significantly enhances structural stability and mitigates long‐term morphological degradation (the bottom row of Figure 4b).

To investigate the interfacial composition and morphological stability after extended cycling, elemental mapping was performed on bare Zn and 4SP2FCD electrodes after 50 cycles (Figure S16). For the bare Zn electrode, no detectable F signal was observed, whereas the 4SP2FCD electrode exhibited a uniformly distributed F signal across the surface, indicating the homogeneous dispersion of FCDs within the SPEEK matrix. Furthermore, the Zn element on the bare Zn electrode displayed a non‐uniform distribution with rough surface morphology and evident polygonal by‐products, as confirmed by the strong and bulk shape S signal, suggesting severe side reactions (basic zinc sulfate precipitation) and poor interfacial stability. In contrast, the 4SP2FCD electrode presented a uniform and dense Zn signal with a significantly suppressed S signal, indicating mitigated side reactions (by‐product: Zn_4_SO_4_(OH)_6_·5H_2_O) and stable Zn deposition. Notably, 4SP2FCD electrode showed a homogeneous C distribution, evidencing the integrity and robustness of the interfacial layer after prolonged cycling. In addition, after 50 cycles, the cross‐sectional SEM image (Figure S17) of the 4SP2FCD interlayer remained intact, with Zn uniformly deposited beneath the interlayer, indicating guided and stable bottom‐up plating, leading to a more condensed and uniform Zn layer with improved cycling stability.

To further investigate the underlying mechanisms, finite element modelling (FEM) simulations using COMSOL were performed to analyse the electric field distribution, Zn^2+^ ion transport, and current density variations at the electrode/electrolyte interface. The deposition process was simulated from 0 to 1000 s, as shown in Figure S18. As shown in the top row of Figure 4c, after 1000 s, the bare Zn electrode exhibits severe electric field distortion near protrusions, while the SPEEK/FCD electrode shows a more uniform field, benefiting from the negatively charged −SO_3_^−^ groups in SPEEK that attract Zn^2+^ and repel SO_4_^2−^, thereby mitigating local field enhancement. In the middle row of Figure 4c, Zn^2+^ ions in the bare Zn electrode accumulate at high electrical‐field sites, promoting tip growth. In contrast, the 4SP2FCD electrode exhibits a more uniform Zn^2+^ distribution, attributed to both chemical and structural regulation from the interlayer. The uniformly embedded FCDs, which refine domain connectivity and homogenize the local field, together with diverse functional groups exhibiting different Zn^2+^ adsorption energies, impart a cation‐perfering ion‐exchange at the interface; fixed anionic sites enrich Zn^2+^ while excluding anions, establishing Zn^2+^‐preferred pathways and a steadier interfacial flux. As shown in the bottom row of Figure 4c, current density on bare Zn is highly localized at protrusions, whereas the SPEEK/FCD layer homogenizes current flow, alleviating interfacial hot spots. These results underscore the multifunctional role of the SPEEK/FCD interlayer in regulating electric field, ion distribution, and current pathways to enable smooth and dendrite‐free Zn deposition.

Previous studies have shown that Zn crystal planes, including Zn (110), Zn (101), and Zn (002), play a crucial role in determining the Zn growth orientation [47]. Among these, the (002) orientation is considered the most favourable for achieving a flatter and more uniform Zn deposition. To assess the Zn growth preference in bare Zn and 4SP2FCD electrodes, the ratio of Zn (002) to Zn (100) peak intensities (*I*_Zn(002)_/*I*_Zn(100)_) in the XRD patterns was analysed. After 10, 50, and 100 cycles, the *I*_Zn(002)_/*I*_Zn(100)_ ratio for the Zn electrode cycled in the 4SP2FCD electrode was found to be 2.96, 4.06, and 1.96, which were significantly higher than those of the bare Zn electrode (0.59, 0.36, and 0.98) under the same conditions (Figure 4d–f). This result suggests that the SPEEK/FCD interlayer strongly promotes the preferential growth of the Zn (002) plane.

### 2.5 Battery Performance Evaluation

**FIGURE 5:**
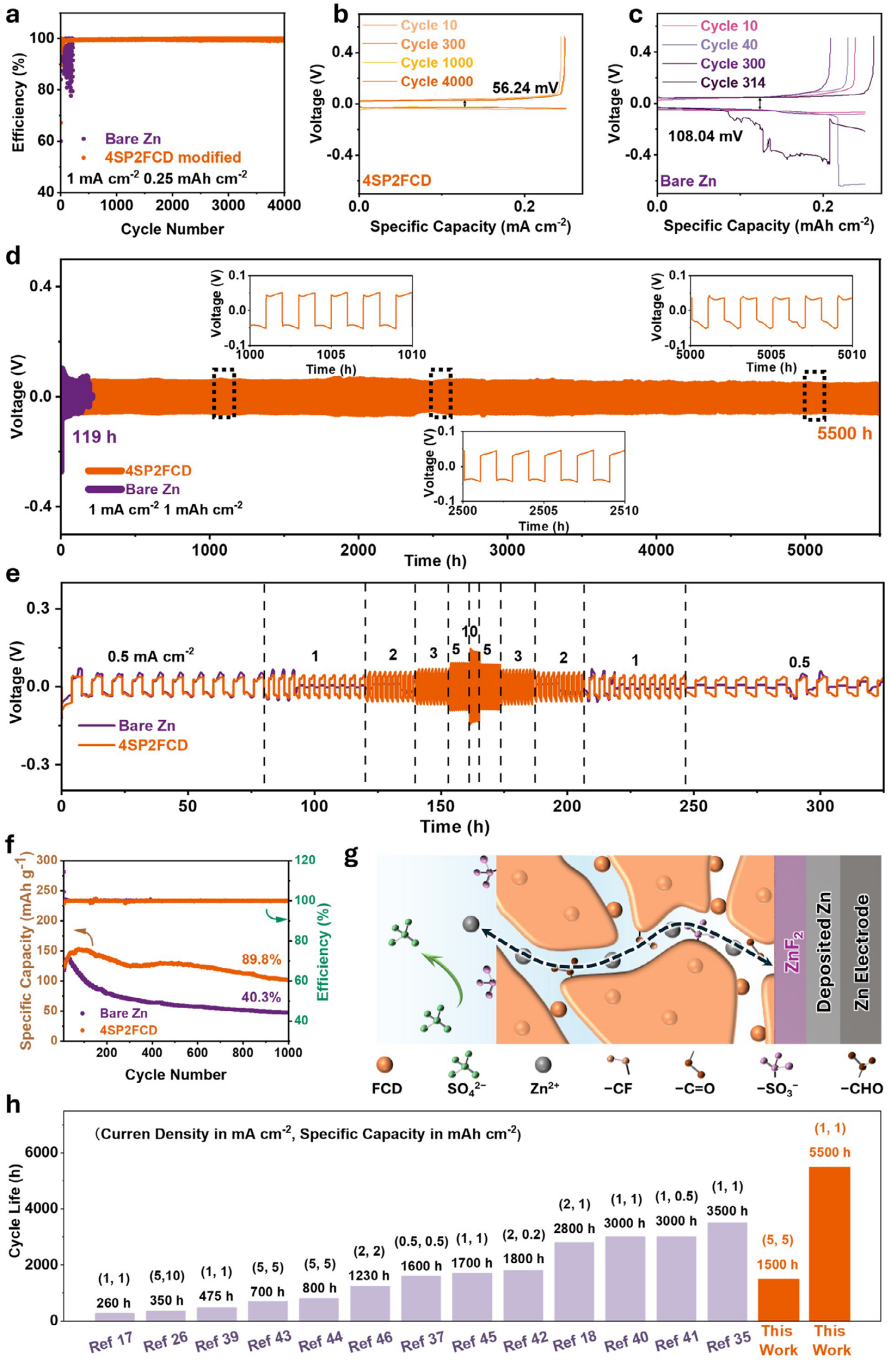
The electrochemical performance. (a) CE of Zn||Cu half‐cells using pristine Zn and 4SP2FCD electrodes at 1 mA cm^−2^, 0.25 mAh cm^−2^. Voltage profiles of Zn||Cu cells at selected cycles using (b) 4SP2FCD and (c) pristine Zn electrodes. (d) Long‐term cycling stability of Zn||Zn symmetric cells at 1 mA cm^−2^, 1 mAh cm^−2^; insets show voltage profiles at 1000, 2500, and 5000 h. (e) Rate performance of Zn||Zn symmetric cells over 0.5–10 mA cm^−2^. (f) Cycling performance of Zn||V_2_O_5_ full cells at 1 A g^−1^. (g) Schematic illustration of the multifunctional role of the SPEEK/FCD interlayer in regulating Zn^2+^ deposition and stabilizing the Zn/electrolyte interface. (h) Comparison of Zn||Zn symmetric cell cycle life and test conditions with previously reported systems [20, 21, 29, 37, 13, 42, 48, 49, 50, 51, 52, 53, 54, 48].

The electrochemical performance of repeated Zn plating/stripping was evaluated in Zn||Cu half‐cells by measuring the Coulombic efficiency (CE). At 1 mA cm^−2^ and 0.25 mAh cm^−2^, the Zn||Cu cell with the 4SP2FCD on both Zn and Cu electrode achieved an average CE of 99.64% after 4000 cycles (Figure 5a), which is much better than that of the bare Zn (declined significantly after just 314 cycles). Furthermore, the overpotential of the cell with the 4SP2FCD electrode is 56.24 mV, and its charge/discharge curves overlap very well even after 4000 cycles. However, the overpotential (108.04 mV) of the cell with a bare Zn electrode was almost twice that of the cell with the 4SP2FCD electrode. The superior CE and low overpotential of the 4SP2FCD electrode are attributed to its good ionic conductivity and excellent suppression of side reactions (Figure 5b,c).

The cycling performance of Zn||Zn symmetric cells with different anodes was evaluated to assess the stability improvements conferred by the SPEEK/FCD interlayer. The 4SP2FCD electrode exhibited significantly prolonged cycling life, achieving 5500 h at 1 mA cm^−2^ and 1 mAh cm^−2^, and 1500 h at 5 mA cm^−2^ and 5 mAh cm^−2^ (Figure 5d; Figure S19), far surpassing the performance of bare Zn. The inset voltage profiles at 1000, 2500, and 5000 h reveal consistent and low polarization over time, further confirming the outstanding interfacial stability and dendrite suppression capability of the 4SP2FCD electrode during long‐term cycling.

In addition, analysis of the rate performance of Zn||Zn symmetric cells across a current density range of 0.5 to 10 mA cm**^−^** ^2^ (Figure 5e) shows the symmetric cell with 4SP2FCD electrode exhibits consistently lower overpotentials, highlighting its role in improving Zn deposition kinetics. Contrastingly, the symmetric cell with a bare Zn electrode suffered a short circuit after only 91 h due to severe dendrite growth. The results indicate the 4SP2FCD interlayer effectively suppresses dendrite formation to promote sustained cycling stability.

To assess the electrochemical performance of the 4SP2FCD electrode in a full cell, cells were assembled with a commercial V_2_O_5_ based cathode and the 4SP2FCD anode. At a current density of 1 A g**^−^** ^1^, the full cell exhibited a peak discharge capacity of 153.27 mAh g**^−^** ^1^ and retained 89.8% of its initial capacity after 1000 cycles (Figure 5f). Comparatively, under the same conditions, the full cell with the bare Zn electrode had a discharge capacity of 126.2 mAh g**^−^** ^1^ and retained only 40.3% of its initial capacity. Additionally, the full cell with the 4SP2FCD electrode displayed significantly improved rate performance compared to the cell with a bare Zn anode (Figure S20). These results demonstrate the critical role of the 4SP2FCD interlayer in enhancing both capacity retention and long‐term cycling stability in AZMBs. Furthermore, as summarized in Table S1 and benchmarked against previously reported CD‐based and polymer‐based Zn anode interlayers (Figure 5h), the 4SP2FCD electrode in this work delivers the most competitive cycling stability and cycling life under both low and high current densities. The ultrathin interlayer thickness of only 600 nm is substantially thinner than most reported counterparts in literature [20, 29, 37, 42, 48, 49, 50, 52, 53, 55].

Collectively, the exceptional performance of 4SP2FCD electrode is primarily attributed to the multifunctional ultra‐thin hybrid SPEEK/FCD interlayer (Figure 5g): (1) The sulfonic acid groups in SPEEK regulate the Zn^2+^ solvation structure, enhancing zinc ion transport and deposition kinetics. (2) The embedded FCDs act as additives that refine the sulfonic acid ion‐exchange domains of the SPEEK matrix by tuning domain size and continuity and enhancing cation selectivity. This delivers a steadier Zn^2+^ flux to the interface and homogenizes the local electric field, lowering the local nucleation current and overpotential and yielding uniform, compact deposition. (3) The fluorine‐rich interfacial environment promotes the in situ formation of a ZnF_2_‐based SEI, which suppresses parasitic reactions and stabilizes the Zn/electrolyte interface, and (4) the hybrid interlayer exhibits excellent mechanical robustness, relieving interfacial stress and effectively suppressing dendrite formation. Based on these synergistic effects, the modified electrode exhibits excellent cyclability, ultra‐long calendar life, excellent mechanical and chemical stability, and outstanding rate performance. It is indicated that the 4SP2FCD electrode is applicable to high‐performance AZMBs applications.

## 3 Conclusion

In summary, an ultra‐thin SPEEK/FCD interlayer is constructed on the surface of Zn metal with significantly improved performance in AZMBs. Through a combination of experimental studies and theoretical calculations, the SPEEK/FCD interlayer is demonstrated to effectively regulate Zn^2+^ solvation, facilitate nucleation, stabilize SEI formation, and accommodate volume fluctuations, contributing to improved electrochemical performance. Raman spectroscopy reveals that the functional groups within the interlayer influence Zn^2+^ solvation, forming an ion transport channel that enhances Zn^2+^ mobility and mitigates side reactions. Nucleation‐overpotential analysis together with DFT calculations indicates that, within the SPEEK/FCD ion‐exchange interlayer, the sulfonic acid groups in SPEEK constitute the primary cation‐selective ion‐exchange domains, while the FCD additives refine these domains (tuning their size/continuity and selectivity). This configuration delivers a steadier Zn^2+^ flux to the interface and homogenizes the local electric field, thereby lowering the nucleation energy barrier and yielding uniform, compact Zn plating. TEM and XPS depth profiling further identify the formation of a ZnF_2_‐rich SEI layer, which stabilizes the Zn‐electrolyte interface and suppresses dendritic growth. Tensile stress measurements highlight the mechanical resilience of the SPEEK/FCD interlayer, demonstrating its ability to withstand volume fluctuations during repeated Zn plating/stripping cycles. Therefore, the Zn||Zn symmetric cells incorporating the 4SP2FCD interlayer exhibit over 5500 h cycle life at 1 mA cm**^−^** ^2^ and 1 mAh cm**^−^** ^2^, and 1500 h at 5 mA cm**^−^** ^2^ and 5 mAh cm**^−^** ^2^, significantly outperforming bare Zn. Zn||V_2_O_5_ full cell with the 4SP2FCD interlayer achieves a peak capacity of 153.27 mAh g**^−^** ^1^ and maintains 89.8% capacity retention after 1000 cycles at 1 A g**^−^** ^1^, demonstrating enhanced long‐term performance. This work provides a simple yet effective interfacial engineering strategy for stabilizing Zn metal anodes, offering new insights into high‐performance and durable Zn metal batteries for practical applications.

## Conflicts of Interest

The authors declare no conflicts of interest.

## Supporting information




**Supporting File**: advs73967‐sup‐0001‐SuppMat.docx.

## Data Availability

The data that support the findings of this study are available from the corresponding author upon reasonable request.;

## References

[1] J. Xu , H. Li , Y. Jin , et al., “Understanding the Electrical Mechanisms in Aqueous Zinc Metal Batteries: From Electrostatic Interactions to Electric Field Regulation,” Advanced Materials 36 (2024): 2309726, 10.1002/adma.202309726. 37962322

[2] M. Song , H. Tan , D. Chao , and H. J. Fan , “Recent Advances in Zn‐Ion Batteries,” Advanced Functional Materials 28 (2018): 1802564, 10.1002/adfm.201802564.

[3] D. Chao , W. Zhou , F. Xie , et al., “Roadmap for Advanced Aqueous Batteries: From Design of Materials to Applications,” Science Advances 6 (2020): aba4098, 10.1126/sciadv.aba4098. PMC7244306 32494749

[4] F. Wang , O. Borodin , T. Gao , et al., “Highly Reversible Zinc Metal Anode for Aqueous Batteries,” Nature Materials 17 (2018): 543–549, 10.1038/s41563-018-0063-z. 29662160

[5] E. Zhao , K. Gao , X. Luo , L. Li , J. Zhao , and H. Li , “Heterostructure VO_2_@VS_2_ Tailored by One‐Step Hydrothermal Synthesis for Stable and Highly Efficient Zn‐ion Storage,” Materials Futures 3 (2024): 045101, 10.1088/2752-5724/ad778d.

[6] Y. Zuo , K. Wang , P. Pei , et al., “Zinc Dendrite Growth and Inhibition Strategies,” Materials Today Energy 20 (2021): 100692, 10.1016/j.mtener.2021.100692.

[7] A. Bayaguud , Y. Fu , and C. Zhu , “Interfacial Parasitic Reactions of Zinc Anodes in Zinc Ion Batteries: Underestimated Corrosion and Hydrogen Evolution Reactions and Their Suppression Strategies,” Journal of Energy Chemistry 64 (2022): 246–262, 10.1016/j.jechem.2021.04.016.

[8] Y. Cui , R. Zhang , S. Yang , L. Liu , and S. Chen , “Research Progress on the Design of Electrolyte Additives and Their Functions for Zinc‐ion Batteries,” Materials Futures 3 (2024): 012102, 10.1088/2752-5724/acef41.

[9] Z. Xiang , Y. Qiu , X. Guo , K. Qi , Z.‐L. Xu , and B. Y. Xia , “Inherited Construction of Porous Zinc Hydroxide Sulfate Layer for Stable Dendrite‐free Zn Anode,” Energy & Environmental Science 17 (2024): 3409–3418, 10.1039/D4EE00721B.

[10] Q. Zhang , J. Luan , Y. Tang , X. Ji , and H. Wang , “Interfacial Design of Dendrite‐Free Zinc Anodes for Aqueous Zinc‐Ion Batteries,” Angewandte Chemie International Edition 59 (2020): 13180–13191, 10.1002/anie.202000162. 32124537

[11] X. Zhang , Y. Liu , S. Wang , et al., “Fundamentals and Design Strategies of Electrolytes for High‐Temperature Zinc‐Ion Batteries,” Energy Storage Materials 70 (2024): 103471.

[12] J. Xu , W. Lv , W. Yang , et al., “In Situ Construction of Protective Films on Zn Metal Anodes via Natural Protein Additives Enabling High‐Performance Zinc Ion Batteries,” ACS Nano 16 (2022): 11392–11404, 10.1021/acsnano.2c05285. 35848633

[13] T.‐B. Song , Z.‐H. Huang , X.‐R. Zhang , J.‐W. Ni , and H.‐M. Xiong , “Nitrogen‐Doped and Sulfonated Carbon Dots as a Multifunctional Additive to Realize Highly Reversible Aqueous Zinc‐Ion Batteries,” Small 19 (2023): 2205558, 10.1002/smll.202205558. 36650986

[14] M. Zhu , X. Li , C. Shi , C. Cai , and J. Zhang , “Recent Research Progress in the Design and Modification of Zinc Metal Anodes for Aqueous Zinc Ion Batteries,” Journal of Energy Storage 101 (2024): 113686, 10.1016/j.est.2024.113686.

[15] T.‐B. Song , Q.‐L. Ma , B.‐J. Wang , X.‐R. Zhang , Y.‐G. Wang , and H.‐M. Xiong , “High‐Capacity and Long‐Life Cathode Constructed Solely by Carbon Dots for Aqueous Zinc‐Ion Batteries,” Angewandte Chemie International Edition 64 (2025): 202503655, 10.1002/anie.202503655. 40546010

[16] Z. Cao , Y. Yang , J. Qin , J. He , and Z. Su , “3D TiO_2_/ZnO Hybrid Framework: Stable Host for Lithium Metal Anodes,” Chemical Engineering Journal 427 (2022): 132026, 10.1016/j.cej.2021.132026.

[17] J. Wu , M. Li , H. Li , Z. Wang , T. Chen , and Y. Wang , “Membranes Constructing with Excellent Performances for Aqueous Zinc‐Ion Battery: A Review,” Coordination Chemistry Reviews 531 (2025): 216478, 10.1016/j.ccr.2025.216478.

[18] C. Fan , W. Meng , and J. Ye , “Towards Advanced Zinc Anodes by Interfacial Modification Strategies for Efficient Aqueous Zinc Metal Batteries,” Journal of Energy Chemistry 93 (2024): 79–110, 10.1016/j.jechem.2023.12.054.

[19] B. Li , Y. Ma , J. Ma , L. Chen , Y. Zhao , and M.‐C. Tang , “Challenges and Opportunities Facing Zinc Anodes for Aqueous Zinc‐Ion Battery,” Energy Materials and Devices 2 (2024): 9370044, 10.26599/EMD.2024.9370044.

[20] M. Liu , J. Cai , H. Ao , Z. Hou , Y. Zhu , and Y. Qian , “NaTi_2_(PO_4_)_3_ Solid‐State Electrolyte Protection Layer on Zn Metal Anode for Superior Long‐Life Aqueous Zinc‐Ion Batteries,” Advanced Functional Materials 30 (2020): 2004885, 10.1002/adfm.202004885.

[21] X. Lv , X. Gu , R. Tian , et al., “Artificial Solid Electrolyte Interphases Stabilized Zn Metal Anodes for High‐Rate and Long‐Lifespan Aqueous Batteries,” Electrochimica Acta 524 (2025): 146053, 10.1016/j.electacta.2025.146053.

[22] H. Wang , G. Zhao , W. Li , S. Watanabe , and X. Wang , “Fluoride‐Based Artificial Interface for a Highly Reversible Zn Metal Anode Interlayer in Aqueous Zinc‐Ion Batteries,” ACS Sustainable Chemistry & Engineering 12 (2024): 355–364, 10.1021/acssuschemeng.3c05796.

[23] X. Lei , Z. Ma , L. Bai , et al., “Porous ZnP Matrix for Long‐Lifespan and Dendrite‐Free Zn Metal Anodes,” Battery Energy 2 (2023): 20230024.

[24] Y. Liang , Y. Wang , H. Mi , et al., “Functionalized Carbon Nanofiber Interlayer Towards Dendrite‐Free, Zn‐Ion Batteries,” Chemical Engineering Journal 425 (2021): 131862, 10.1016/j.cej.2021.131862.

[25] C. Zhou , L. Shan , Q. Nan , et al., “Construction of Robust Organic–Inorganic Interface Layer for Dendrite‐Free and Durable Zinc Metal Anode,” Advanced Functional Materials 34 (2024): 2312696, 10.1002/adfm.202312696.

[26] B. M. Mahimai , G. Sivasubramanian , K. Sekar , D. Kannaiyan , and P. Deivanayagam , “Sulfonated Poly(ether ether ketone): Efficient Ion‐Exchange Polymer Electrolytes for Fuel Cell Applications–a Versatile Review,” Materials Advances 3 (2022): 6085–6095, 10.1039/D2MA00562J.

[27] F. T. Chikumba , M. Tamer , L. Akyalçın , and S. Kaytakoğlu , “The Development of Sulfonated Polyether Ether Ketone (sPEEK) and Titanium Silicon Oxide (TiSiO_4_) Composite Membranes for DMFC Applications,” International Journal of Hydrogen Energy 48 (2023): 14038–14052, 10.1016/j.ijhydene.2022.12.293.

[28] T. G. Habteyes , E. R. Westphal , K. M. Plackowski , et al., “Hierarchical Self‐Assembly of Carbon Dots into High‐Aspect‐Ratio Nanowires,” Nano Letters 23 (2023): 9474–9481, 10.1021/acs.nanolett.3c02977. 37831934

[29] H. Fan , M. Wang , Y. Yin , et al., “Tailoring Interfacial Zn^2+^ Coordination via a Robust Cation Conductive Film Enables High Performance Zinc Metal Battery,” Energy Storage Materials 49 (2022): 380–389, 10.1016/j.ensm.2022.04.031.

[30] M. Yi , M. Jing , Y. Yang , et al., “Recent Developments of Carbon Dots for Advanced Zinc‐Based Batteries: A Review,” Advanced Functional Materials 34 (2024): 2400001, 10.1002/adfm.202400001.

[31] Z. Ebrahim Nataj , A. S. Kazemi , and Y. Abdi , “Surface Effects and Wettability Measurement Considerations in Fluorinated Carbon Nanotubes,” Applied Physics A 127 (2021): 874, 10.1007/s00339-021-05029-z.

[32] Y. Fan , D. Tongren , and C. J. Cornelius , “The Role of a Metal Ion Within Nafion Upon Its Physical and Gas Transport Properties,” European Polymer Journal 50 (2014): 271–278, 10.1016/j.eurpolymj.2013.11.011.

[33] T. Agarwal , A. K. Prasad , S. G. Advani , S. K. Babu , and R. L. Borup , “Infrared Spectroscopy for Understanding the Structure of Nafion and Its Associated Properties,” Journal of Materials Chemistry A 12 (2024): 14229–14244, 10.1039/D3TA05653H.

[34] Z. Miao , F. Zhang , H. Zhao , et al., “Tailoring Local Electrolyte Solvation Structure via a Mesoporous Molecular Sieve for Dendrite‐Free Zinc Batteries,” Advanced Functional Materials 32 (2022): 2111635, 10.1002/adfm.202111635.

[35] Y. Du , K. Sun , L. Zheng , Y. Xie , S. Liao , and T. Wei , “Laser‐Induced Fluorine‐Containing Coating on Zinc Anode Surface for Regulated Zn (002) Deposition,” Materials Chemistry Frontiers 9 (2025): 1383–1388.

[36] A. Lejarazu‐Larrañaga , E. Sánchez‐Díez , Y. Zhang , et al., “Tuning the Ion‐Transport Nanochannels of Sulfonated Poly (Ether Ether Ketone) Membranes for Efficient Aqueous Organic Redox Flow Battery,” Energy Materials 5 (2025): 500044.

[37] Y. Wang , Y. Wang , C. Chen , et al., “Optimizing the Sulfonic Groups of a Polymer to Coat the Zinc Anode for Dendrite Suppression,” Chemical Communications 57 (2021): 5326–5329, 10.1039/D1CC00924A. 33942821

[38] B. Song , X. Wang , H. Gao , W. Gao , and X. Ma , “Uniform Zinc‐Ion Deposition Regulated by Thin Sulfonated Poly(Ether Ketone) Layer for Stabilizing Zn Anodes,” Nanotechnology 35 (2023): 025401, 10.1088/1361-6528/ad0245. 37820634

[39] X. Xie , L. Deng , L. Li , A. Pan , S. Liang , and G. Fang , “Modulating Interfacial Zn^2+^ Deposition Mode towards Stable Zn Anode via Bimetallic Co‐Doped Coating,” Energy Storage Materials 73 (2024): 103834, 10.1016/j.ensm.2024.103834.

[40] Y. Ren , H. Bian , S. Chang , et al., “Promoting Adsorption‐Diffusion‐Deposition of Zn^2+^ via a Highly Reactive Modulator for Dendrite‐Free and Kinetics‐Enhanced Zn Metal Anode,” Chemical Engineering Journal 498 (2024): 155245, 10.1016/j.cej.2024.155245.

[41] C. Chang , S. Hu , T. Li , et al., “A Robust Gradient Solid Electrolyte Interphase Enables Fast Zn Dissolution and Deposition Dynamics,” Energy & Environmental Science 17 (2024): 680–694, 10.1039/D3EE03422D.

[42] Z. Ge , L. Xu , Y. Xu , et al., “Multifunctional Fluorinated Carbon Dots Artificial Interface Layer Coupled With In‐Situ Generated Zn^2+^ Conductor Interlayer Enable Ultra‐Stable Zn Anode,” Nano Energy 119 (2024): 109053, 10.1016/j.nanoen.2023.109053.

[43] H. Wang , Y. Chen , H. Yu , et al., “A Multifunctional Artificial Interphase with Fluorine‐Doped Amorphous Carbon Layer for Ultra‐Stable Zn Anode,” Advanced Functional Materials 32 (2022): 2205600, 10.1002/adfm.202205600.

[44] C. Chen , R. Guo , S. Ganapathy , et al., “Enhancing Zn Deposition Reversibility on MXene Current Collectors by Forming ZnF_2_‐Containing Solid‐Electrolyte Interphase for Anode‐Free Zinc Metal Batteries,” Small (2025): 2407226, 10.1002/smll.20.2407226. PMC13137241 39871739

[45] L. Ma , Q. Li , Y. Ying , et al., “Toward Practical High‐Areal‐Capacity Aqueous Zinc‐Metal Batteries: Quantifying Hydrogen Evolution and a Solid‐Ion Conductor for Stable Zinc Anodes,” Advanced Materials 33 (2021): 2007406, 10.1002/adma.202007406. 33604973

[46] Y. Yang , C. Liu , Z. Lv , et al., “Synergistic Manipulation of Zn^2+^ Ion Flux and Desolvation Effect Enabled by Anodic Growth of a 3D ZnF_2_ Matrix for Long‐Lifespan and Dendrite‐Free Zn Metal Anodes,” Advanced Materials 33 (2021): 2007388, 10.1002/adma.202007388. 33554430

[47] R. Deng , Z. He , F. Chu , et al., “An Aqueous Electrolyte Densified by Perovskite SrTiO_3_ Enabling High‐voltage Zinc‐ion Batteries,” Nature Communications 14 (2023): 4981, 10.1038/s41467-023-40462-z. PMC10435537 37591851

[48] J. Wang , Y. Yu , R. Chen , et al., “Induced Anionic Functional Group Orientation‐Assisted Stable Electrode‐Electrolyte Interphases for Highly Reversible Zinc Anodes,” Advanced Science 11 (2024): 2402821, 10.1002/advs.202402821. 38666375 PMC11220644

[49] Q. Jian , Y. Wan , Y. Lin , M. Ni , M. Wu , and T. Zhao , “A Highly Reversible Zinc Anode for Rechargeable Aqueous Batteries,” ACS Applied Materials & Interfaces 13 (2021): 52659–52669, 10.1021/acsami.1c15628. 34723460

[50] H. Zhang , S. Li , L. Xu , et al., “High‐Yield Carbon Dots Interlayer for Ultra‐Stable Zinc Batteries,” Advanced Energy Materials 12 (2022): 2200665, 10.1002/aenm.202200665.

[51] H. Zhang , R. Guo , S. Li , et al., “Graphene Quantum Dots Enable Dendrite‐free Zinc Ion Battery,” Nano Energy 92 (2022): 106752, 10.1016/j.nanoen.2021.106752.

[52] H. Fan , M. Li , and E. Wang , “Anion‐Functionalized Interfacial Layer for Stable Zn Metal Anodes,” Nano Energy 103 (2022): 107751, 10.1016/j.nanoen.2022.107751.

[53] W. Dong , C. Liu , X. Ji , et al., “Construction of Cation‐Sieving Function Layers Enabling Dendrite‐Free Zinc Metal Anodes for Durable Aqueous Systems,” Small Methods 8 (2024): 2300799, 10.1002/smtd.202300799. 37728187

[54] Q. He , G. Fang , Z. Chang , et al., “Building Ultra‐Stable and Low‐Polarization Composite Zn Anode Interface via Hydrated Polyzwitterionic Electrolyte Construction,” Nano‐Micro Letters 14 (2022): 93, 10.1007/s40820-022-00835-3. 35384517 PMC8986915

[55] Z. Jiang , Z. Du , R. Pan , et al., “Electrosynthesis of Metal–Organic Framework Interlayer to Realize Highly Stable and Kinetics‐Enhanced Zn Metal Anode,” Advanced Energy Materials 14 (2024): 2402150, 10.1002/aenm.202402150.

