# Supplementary material for: Ultra‐Thin Hybrid Ion Exchange Interlayer for High Performance Aqueous Zn Metal Batteries
Source: Adv Sci (Weinh). 2026 Jan 23;13(18):e22394. doi: 10.1002/advs.202522394 (PMC13042568; doi:10.1002/advs.202522394)
Supplement: Supplementary file 1 — Supporting File: advs73967‐sup‐0001‐SuppMat.docx. [file ADVS-13-e22394-s001.docx]

Supporting Information for

Ultra-thin Hybrid Ion Exchange Interlayer for High Performance Aqueous Zn Metal Batteries

Tong Yang, Weijia Meng, Tan Trung Kien Huynh, Zhengyu Wang, Jiaye Ye*, Yang Yang, Minh Tam Hoang, Zixuan Liu, Zijian Cai, Meiqing Guo*, Jingsan Xu, Hongxia Wang*

T. Yang, T. T. K. Huynh, J. Ye, Y. Yang, M. T. Hoang, Z. Liu, Z. Cai, J. Xu, H. Wang

School of Chemistry and Physics, Queensland University of Technology, 2 George Street, Brisbane City QLD 4000, Australia

E-mail: hx.wang@qut.edu.au (*H. Wang*); jiaye.ye@qut.edu.au (*J. Ye*)

W. Meng

Shaanxi Key Laboratory of New Transportation Energy and Automotive Energy Saving, School of Energy and Electrical Engineering, Chang'an University, Xi'an 710061, Shaanxi, China

Z. Wang, M. Guo

Institute of Applied Mechanics, College of Aeronautics and Astronautics, Taiyuan University of Technology, Taiyuan 030024, China

E-mail: guomeiqing@tyut.edu.cn (*M. Guo*)

**Experimental Section**

**Materials:** Poly(ether ether ketone) (PEEK), concentrated sulfuric acid (H_2_SO_4_, 98wt.%), acetaldehyde (≥99.5%), 4-fluorobenzaldehyde (≥98%), dimethylformamide (DMF, anhydrous, 99.8%), zinc sulfate heptahydrate (ZnSO_4_·7H_2_O, ACS reagent, 99%), poly(vinylidene fluoride) (PVDF, average Mw ~534,000 by GPC), and 1-Methyl-2-pyrrolidinone (NMP, anhydrous, 99.5%) were purchased from Sigma-Aldrich and used as received. Super P carbon black was obtained from IMERYS group.

**Synthesis of sulfonated poly(ether ether ketone):** To trade off the ionic conductivity and stability of the polymer, the degree of sulfonation of the sulfonated poly(ether ether ketone) (SPEEK) was approximately 70%. In a typical procedure, 16 g of poly(ether ether ketone) powder was slowly added to 200 mL of concentrated sulfuric acid (H_2_SO_4_, 98 wt.%) under constant stirring at 65°C for 50 minutes. Then, the polymer was washed multiple times with deionized water until the pH of the filtrate turning to neutral. The obtained SPEEK was dried under vacuum at 60°C for 24 h before further characterization and use.

**Synthesis of carbon dots and fluorinated carbon dots:** Carbon dots (CD) were synthesized via an aldol condensation reaction ^[1]^. 3 g of NaOH was dissolved in 5 mL of deionized water under stirring. The solution was then slowly added dropwise into 10 mL of acetaldehyde (40% aqueous solution) under continuous stirring. The reaction mixture was left to stand at room temperature for 120 h to facilitate the formation of carbon dots. After the reaction, the solution was sonicated with diluted hydrochloric acid and DI water until it became a flocculant. Subsequently, the solution was washed with DI water and filtered to obtain the CD. Finally, the obtained CD was dried in a vacuum oven at 40°C for 12 h. The synthesis of fluorinated carbon dots (FCD) was conducted in the same method as CD synthesis, except that an additional 5 mL of 4-fluorobenzaldehyde was added as a source of fluorine.

**Fabrication of modified zinc metal anodes:** Under room temperature, uniform dispersions of SPEEK of different weight percentages were obtained by dissolving SPEEK into DMF. Next, different weights of CD or FCD were added to the SPEEK dispersions and continuously stirred to form various uniform mixtures of SPEEK/CD or SPEEK/FCD. The Zn foil was pretreated with the UV Ozone to improve the wettability. Then, these mixtures were spin-coated on the Zn foil (thickness: 100 µm) to form uniform thin films, yielding modified Zn electrodes covered with SPEEK/(F)CD. These modified Zn electrodes were named as *x*SP*y*FCD electrodes, with SP referring to SPEEK, *x* representing the number of the weight percentage of SPEEK in the dispersion (*x =* 1, 2, 4, 10, and 20 wt.%), and *y* indicating the number of the weight percentage of CD or FCD to the SPEEK (y = 0.25, 0.5, 1, 2, 4, 6, and 8 wt.%). For example, 4SP2FCD represents this modified Zn electrode prepared by coating the zinc foil surface with a mixture containing 4 wt.% SPEEK and 2 wt.% FCD, while 4SP2CD is obtained by coating the zinc foil surface with a mixture containing 4 wt.% SPEEK and 2 wt.% CD. After coating, the modified zinc foil was transferred to a vacuum oven and dried at 60 °C for 24 h. Finally, the coated Zn foil was punched into 12 mm diameter discs.

**Preparation of ZnSO_4_ electrolyte:** Deionized water was purged with nitrogen gas to eliminate dissolved oxygen. A 2 mol L^−1^ ZnSO_4_ electrolyte was prepared by dissolving ZnSO_4_·7H_2_O in the pre-purged deionized water under continuous stirring to form a homogeneous solution. The electrolyte was equilibrated at room temperature for 12 h before use to ensure complete dissolution and stability. The final electrolyte solution was filtered through a 0.22 μm filter to remove any undissolved particles.

**Preparation of vanadium oxide cathode:** The vanadium oxide (V_2_O_5_) cathode was prepared using a slurry coating method. V_2_O_5_, PVDF, and Super P carbon were mixed in an 8:1:1 weight ratio, ground for 30 minutes to ensure uniform dispersion, and subsequently combined with the 400 µl NMP solution to form a cathode ink. The resulting slurry was coated on a stainless-steel foil by a doctor blade, ensuring a uniform layer. The coated electrodes were then dried at 80 °C overnight in a vacuum oven to remove residual solvent. After drying, the coated foil was punched into 12 mm diameter discs, and the final V_2_O_5_ mass loading for full-cell testing was approximately 1 mg cm^-2^.

**Material Characterization**

The morphologies of Zn anode materials were observed using scanning electron microscopy (SEM, JEOL7001). The crystalline structures of all Zn electrodes and Cu electrodes were analyzed by X-ray diffraction (XRD, Rigaku Smart Lab XRD). Raman spectra were collected using a Renishaw Qontor Raman microscope with a 532 nm excitation laser. The surface chemical states were examined via X-ray photoelectron spectroscopy (XPS, Kratos Axis Supra), and an argon gas cluster ion source was used for depth profiling of the Zn electrode surface. In-situ optical microscopy images of transparent cells were obtained using a Leica M125 Zoom Stereo Microscope. Zeta potential measurements were conducted using a Malvern Zetasizer Nano ZS. Additionally, Fourier-transform infrared spectroscopy (FTIR, Bruker Tensor 27 FTIR Spectrometer) was used to measure the functional groups present in the interlayer materials. The ZnSO_4_ electroyte contact angle measurements were conducted using a Biolin Scientific Attension Theta optical tensiometer. Tensile stress testing was carried out using an Instron 6800 universal testing machine with 6 mm min^–1^ tensile speed.

The Zn^2+^ transference number was evaluated using Zn||Zn symmetric cells with different electrolytes by combining electrochemical impedance spectroscopy (EIS) and chronoamperometry (CA). The value was calculated according to the standard transference number equation.

$\text{t}_{\text{Zn}^{\text{2+}}}\text{=}\frac{\text{I}_{\text{s}}\text{(∆V}-\text{I}_{\text{0}}\text{R}_{\text{0}}\text{)}}{\text{I}_{\text{0}}\text{(∆V}-\text{I}_{\text{s}}\text{R}_{\text{s}}\text{)}}$………………….(1)

Here, *I*_0_ and *R*_0_ represent the initial current and interfacial resistance, respectively, while *I*ₛ and *R*ₛ correspond to the steady-state values. The applied voltage is denoted as Δ*V*. The desolvation energy (*E*ₐ) for Zn deposition in different electrolytes was determined using EIS measurements on Zn||Zn symmetric cells over a range of temperatures. *E*ₐ was extracted based on the Arrhenius equation:

$\frac{\text{1}}{\text{R}_{\text{ct}}}\text{ = A}\text{e}^{\text{(}-\text{-}\frac{\text{E}_{\text{a}}}{\text{R}\text{T}}\text{)}}$……………………(2)

here, *R*_ct_ denotes the charge transfer resistance extracted from EIS spectra, *R* is the universal gas constant, and *T* represents the absolute temperature.

The ionic conductivities of SPEEK and SPEEK/FCD were calculated using the equation below.

$\text{σ = }\frac{\text{L}}{\text{R}\text{×}\text{S}}$……………………..(3)

here, *R* is the resistance obtained from EIS measurements of Zn||Zn symmetric cells with various electrolytes, *L* is the separator thickness, and *S* denotes the electrode–electrolyte contact area.

**Electrochemical Measurement:**

CR2032-type coin cells were assembled to evaluate the electrochemical performance of ZMBs. Glass microfiber filters (Whatman GF/A 1820-55) were used as separators. The galvanostatic discharge/charge cycling tests for Zn||Zn symmetric cells, Zn||V_2_O_5_ full cells, and Zn||Cu half-cells were conducted using a Neware multichannel galvanostatic tester.

Linear sweep voltammetry (LSV), Tafel plots, nucleation overpotential (NOP), chronoamperometry (CA), and electrochemical impedance spectroscopy (EIS) measurements were performed using a VMP-300 potentiostat. LSV, Tafel plots, and NOP measurements were conducted in Zn||Zn symmetric cells. Tafel plots were recorded by scanning between −1.5 V and 1.5 V at a scan rate of 2 mV s^−1^. Chronoamperometry (CA) measurements were carried out in Zn||Zn symmetric cells under a fixed overpotential of 20 mV.

**Density Functional Theory** **Calculation (DFT) Method**

The calculation was performed using the projector-augmented wave (PAW) method of the Vienna ab initio simulation package (VASP) based on DFT ^[2, 3]^. The electron interactions were described using GGA (generalized-gradient approximation) with the PBE functional (Perdew-Burke-Ernzerhof). An on-site Hubbard-like interaction in the PBE-GGA method augments the standard DFT energy function ^[4, 5]^. The computationally cost-effective vdw-DF3 functional for taking vdW interactions into account to get a better description of weak physical interactions. In the vertical direction, a vacuum layer of at least 15 Å in thickness is introduced for all the surfaces and interfaces for the adsorption model. The width of smearing was chosen as 0.05 eV, and the k-point sampling was restricted to the Gamma point only. The kinetic energy cutoff was set to 450 eV. The convergence criterion was set to 10^−5^ eV and 0.03 eV/Å for the energy and maximum force for the optimization. Quantum chemistry calculations parts were performed using the Gaussian 09 package. Geometrical optimization and frequency analysis adopted the B3LYP method with 6-311G* basis sets. Weak intermolecular interactions are described using GD3BJ. The electrostatic surface potential (ESP) was carried out based on DFT.

**COMSOL Calculation Method**

This study utilized the "Diluted Species Transport" and "Current Distribution" modules of COMSOL Multiphysics software to simulate the electric field intensity and Zn^2+^ ion concentration at the electrode-electrolyte interface. Reaction boundary conditions were applied to the upper and lower boundaries of the geometric region. The Butler-Volmer equation was used to describe the reaction kinetics at the cathode and anode, while the Nernst-Planck equation described the diffusion and electromigration of charged ions. In the simplified 2D model cell, the dimensions were set to 5 μm in length and 4 μm in width, with humps (0.5 μm in height) representing the initial Zn grains. The rectangular array, with a height of 0.5 μm and a width of 0.1 μm, was depicted as the PSF/AG with a finger-like porous structure. The anode (the lower boundary of the model) was set to be grounded (with a potential of 0 V). The initial concentration of cations and anions is set to 2 M.

The mathematical description of the Nernst-Planck equation is:

$\text{N}_{\text{i}}\text{ }\text{= }-\text{D}_{\text{i}}\text{∇}\text{c}_{\text{i}}-\text{μ}_{\text{i}}\text{c}_{\text{i}}\text{∇}\text{φ }\text{, (}\text{i}\text{ }\text{= 1, 2, 3….)}$………………….(4)

where, *N_i_* and *D_i_* represent the flux and diffusion coefficient of a specific ion, with *i* indicating the component. *µ_i_*, *c_i_*, and *φ* represent the ion mobility, ion concentration, and electrolyte potential, respectively. Additionally, the law of mass conservation and the condition of electroneutrality are ensured by the following equations:

$\frac{\text{∂}\text{c}_{\text{i}}}{\text{∂}\text{t}}\text{ }\text{= }-\text{∇}\text{N}_{\text{i}}\text{ ( }\text{i}\text{ = 1, 2, 3….)}$………………….……….(5)

$\text{0 = }\sum\text{i}\text{z}_{\text{i}}\text{c}_{\text{i}}$………………………………………..…….(6)

The geometric model used in the COMSOL simulation is shown in Figure S1.


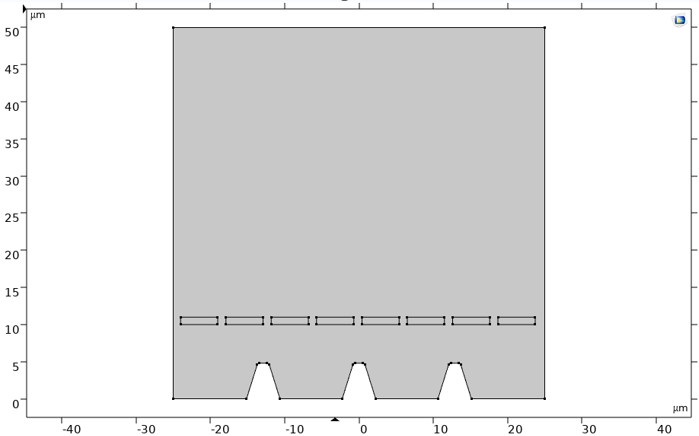


**Figure S1.** Schematic of the COMSOL simulation module constructed to evaluate the Zn^2+^ transport behavior across the interlayer-modified electrode surface.


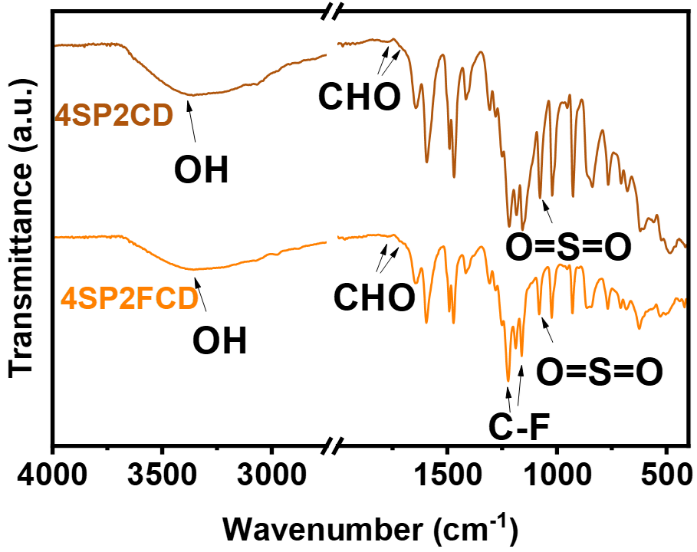


**Figure S2.** The FTIR spectra of 2 mol L^−1^ ZnSO_4_ electrolyte on (a) 4SP2CD electrode, and (b) 4SP2FCD electrode.


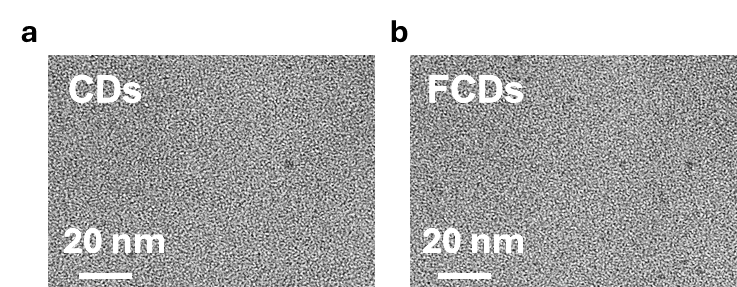


**Figure S3.** TEM images of (a) CDs and (b) FCDs.

We first optimized the ratio between SPEEK and the CD additive. Initial charge/discharge profiles of Zn||Zn symmetric cells (Figure. S4) were used to evaluate the oxidation and reduction overpotential. The SPEEK coating significantly reduced polarization compared to bare Zn (–0.224 V / 0.080 V vs. –0.271 V / 0.080 V), indicating improved Zn^2+^ kinetics. Further incorporation of CDs showed that both the bare SPEEK (1SP) and the 2:1 SPEEK-to-CD formulation (1SP0.5CD) effectively enhanced interfacial behavior, with 1SP0.5CD exhibiting the narrowest potential window (–0.200 V / 0.068 V). For the bare SPEEK film, ion-transport pathways are mainly constructed by continuously distributed sulfonic groups, forming fully open and low-resistance channels. Upon introducing a moderate amount of CDs, these pathways are partially refined rather than completely blocked, leading to slightly increased transport resistance while still maintaining effective Zn^2+^ migration. However, higher CDs content increased overpotential, likely due to ion transport obstruction by embedded CDs. Based on these results, bare SPEEK and the 2:1 formulation were identified as the most promising configurations, upon which further optimization via thickness regulation was conducted.


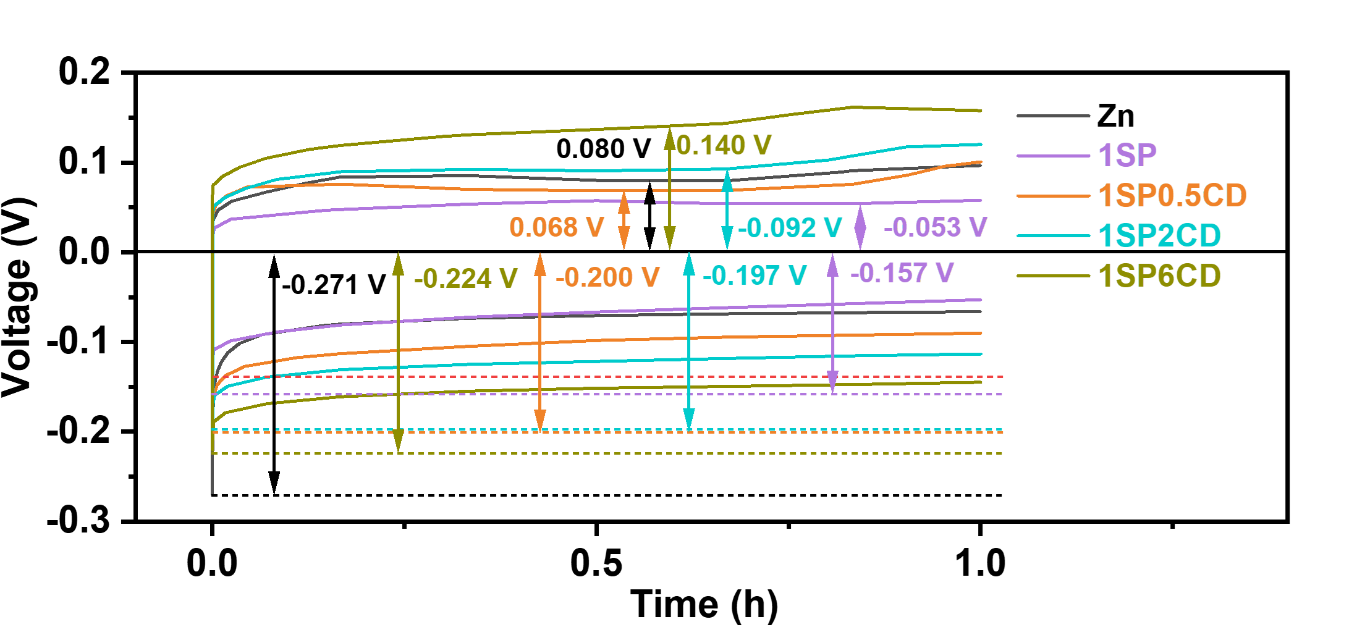


**Figure S4.** The oxidation and reduction overpotential of the Zn electrodes modified with various ratios of SPEEK and FCDs were evaluated with Zn||Zn symmetric cell at 1 mA cm^-2^ and 1 mAh cm^-2^.

Building on the optimal 2:1 SPEEK-to-CD ratio, we tuned the SPEEK concentration to control interlayer thickness. Voltage profiles (Figure. S5) reveal that 4SP2CD (4% SPEEK and 2% CDs) exhibited the smallest polarization (0.053 V /–0.162 V), indicating reduced interfacial resistance and improved Zn plating/stripping kinetics. This composition was identified as the most effective formulation.


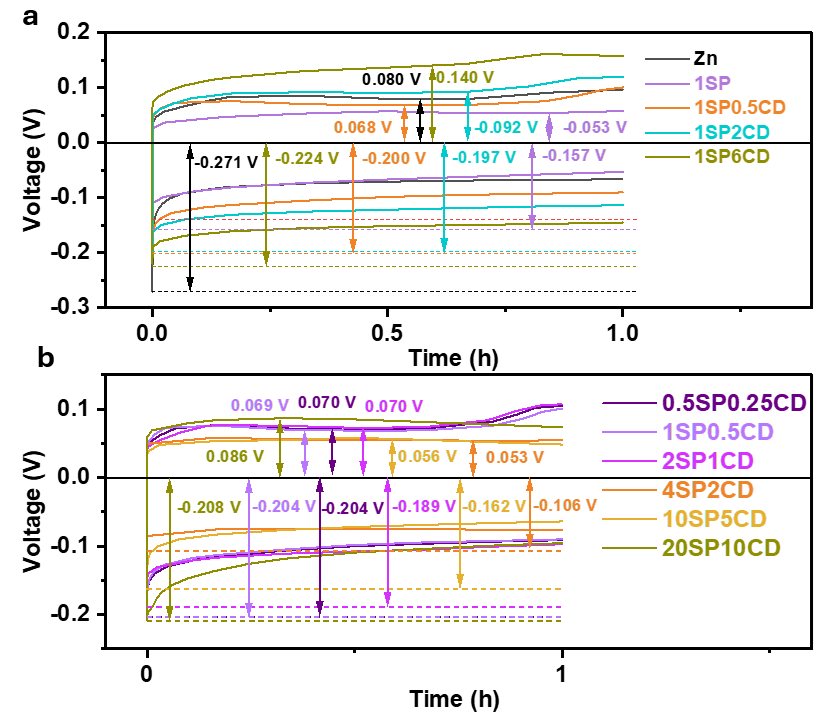


**Figure S5.** Voltage profiles of Zn electrodes modified with varied SPEEK/FCD ratios, tested at 1 mA cm^-2^ and 1 mAh cm^-2^. The 4SP2CD sample displayed the lowest overpotential (0.053 V /−0.162 V), highlighting its superior Zn^2+^ plating/stripping reversibility compared to other tested formulations.


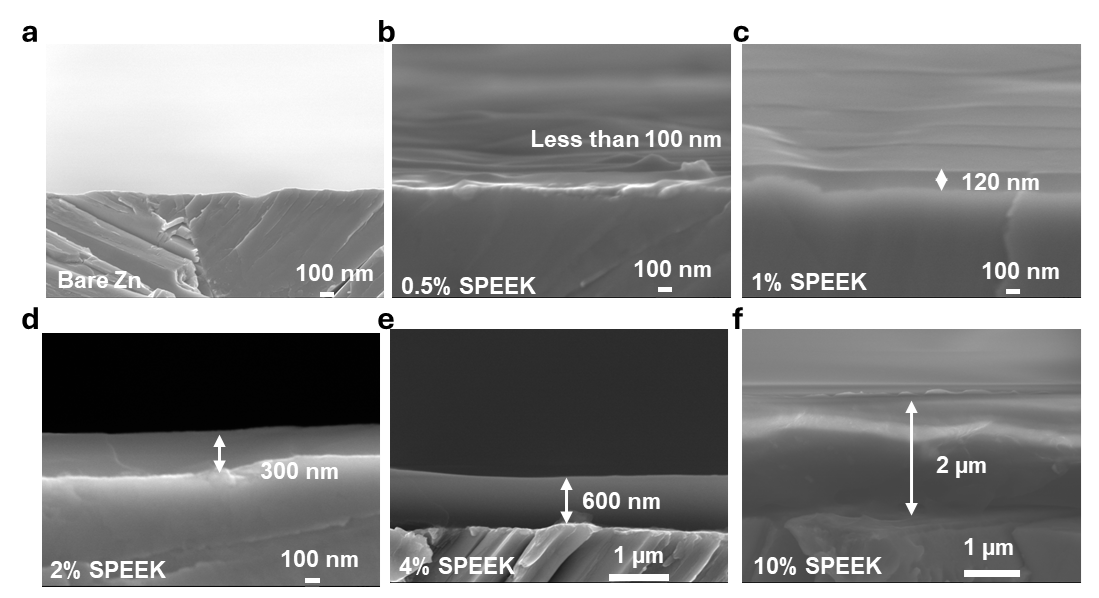


**Figure S6.** Cross-sectional SEM images of Zn electrodes coated with different concentrations of SPEEK: (a) bare Zn, (b) 0.5%, (c) 1%, (d) 2%, (e) 4%, and (f) 10%.


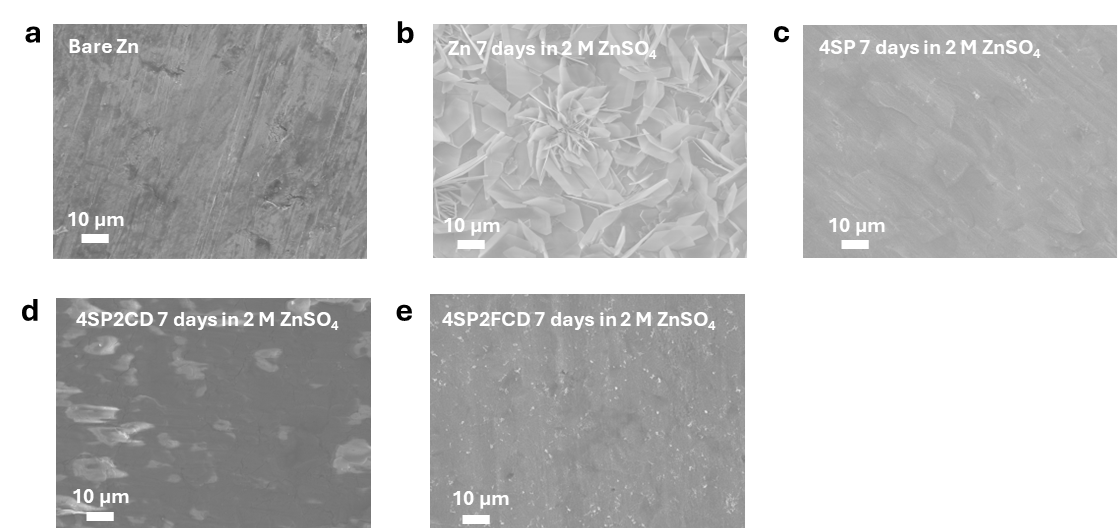


**Figure S7.** The SEM images of (a) initial bare Zn, and (b) bare Zn, (c) 4SP, (d) 4SP2CD, and (e) 4SP2FCD electrodes being immersed in 2 mol L^−1^ after 7 days.


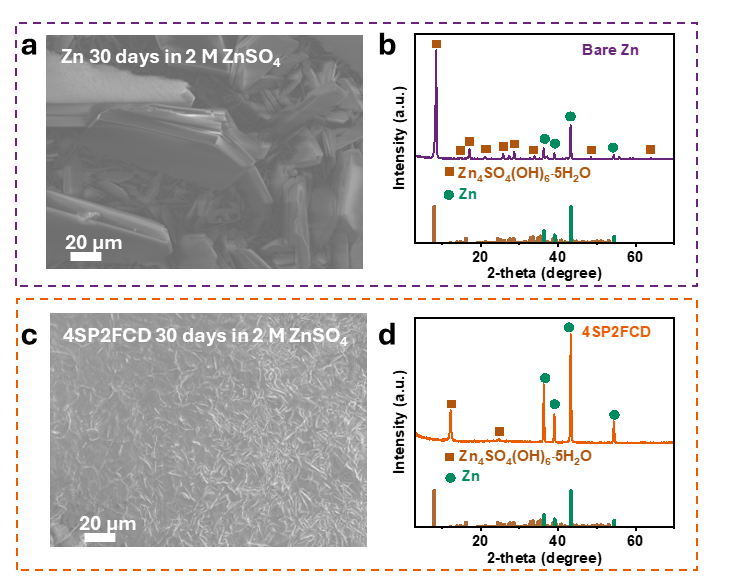


**Figure S8.** Immersion stability test of bare Zn and 4SP2FCD electrodes. (a) SEM images of bare Zn after 30 days of immersion, showing the formation of loose by-products with increasing size, reaching ~100 μm in diameter. (b) XRD patterns of bare Zn before and after immersion, confirming the presence of newly formed by-products. (c) SEM images of 4SP2FCD electrode after 30 days of immersion, revealing a smooth surface with a stable protective film. (d) XRD patterns of 4SP2FCD electrode, demonstrating that the interlayer maintains structural integrity over prolonged exposure.


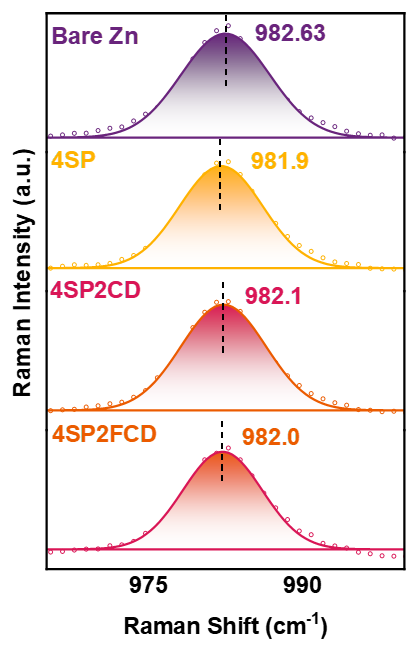


**Figure S9.** Raman spectra showing shifts in SO_4_^2−^.


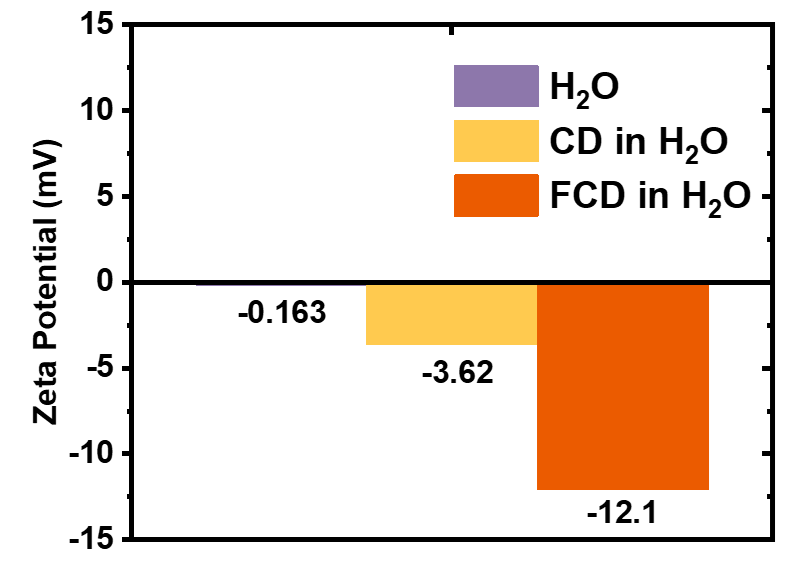


**Figure S10.** Zeta potential measurements of CDs and FCDs in water.


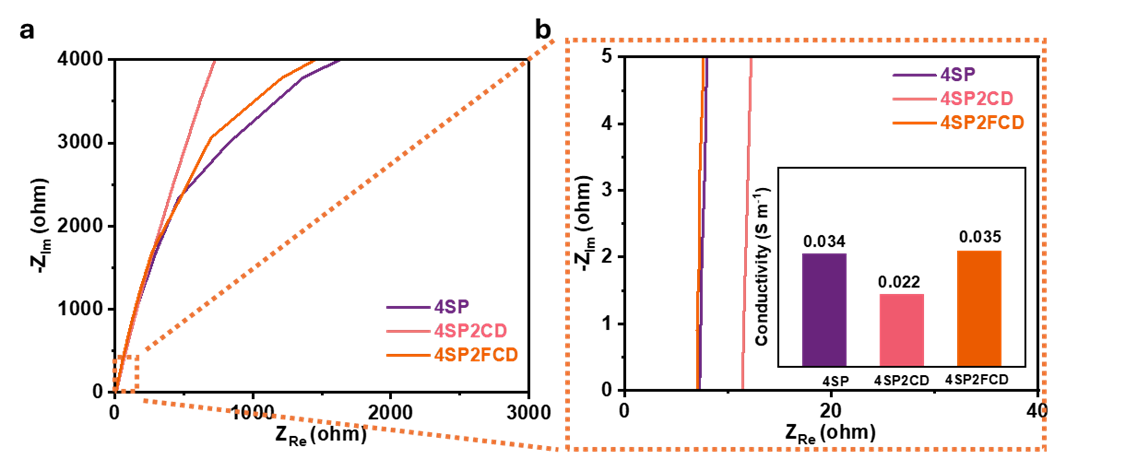


**Figure S11.** Large scale (a) and small scale of (b) Nyquist plots and ionic conductivity of 4SP, 4SP2CD, and 4SP2FCD interlayers. (inset: ionic conductivity of different electrode)

**
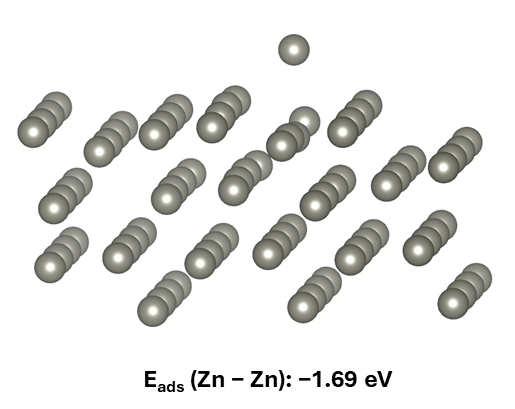
**

**Figure S12.** Optimized atomic configuration of the Zn (101) surface used for density functional theory (DFT) calculations.


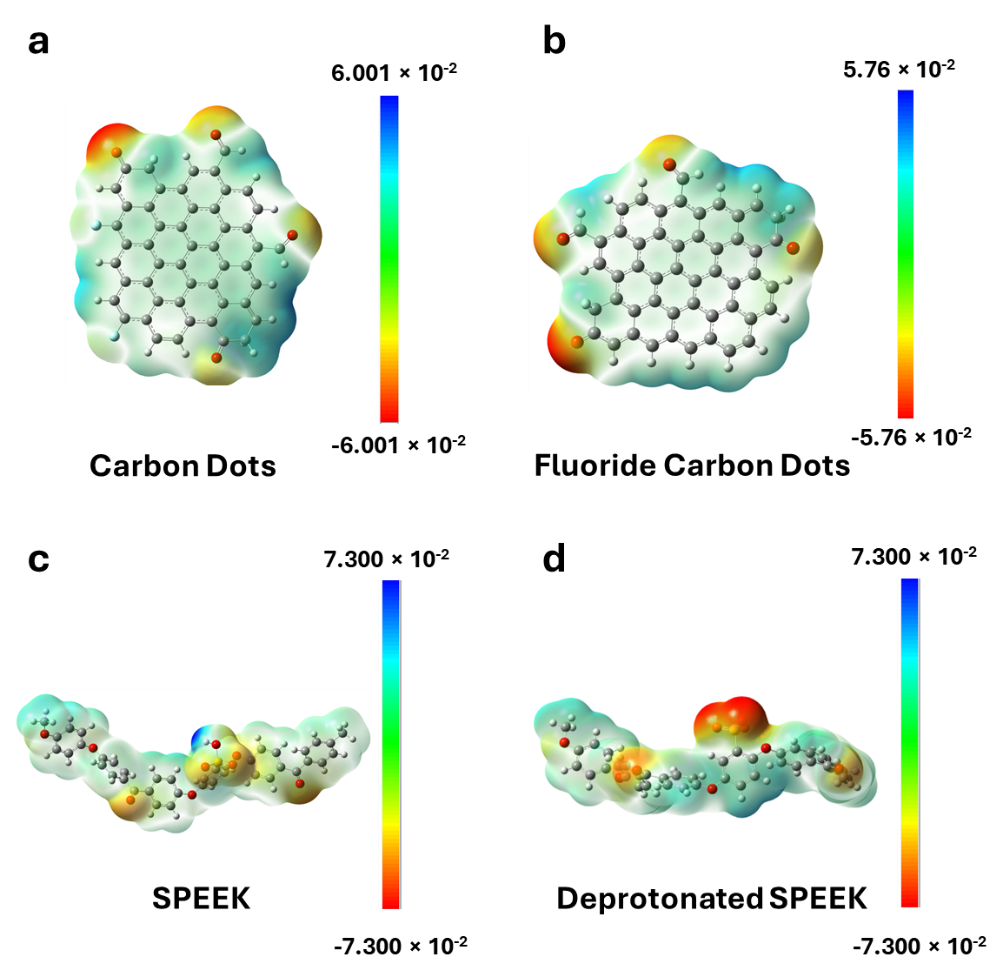


**Figure S13.** Electrostatic potential maps of (a) carbon dots, (b) fluorinated carbon dots, (c) SPEEK, and (d) deprotonated SPEEK. Red and blue regions indicate areas of negative and positive electrostatic potential, respectively. The distinct surface charge distributions highlight the role of functional groups in influencing Zn^2+^ adsorption behavior and interfacial ion interactions.


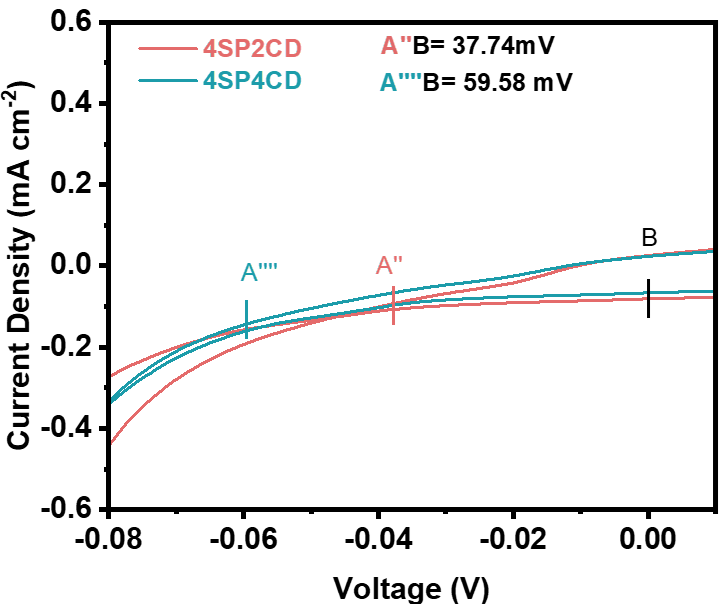


**Figure S14.** Nucleation overpotential (NOP) curves of Zn symmetric cells with 4SP2CD electrode and 4SP4CD electrode.


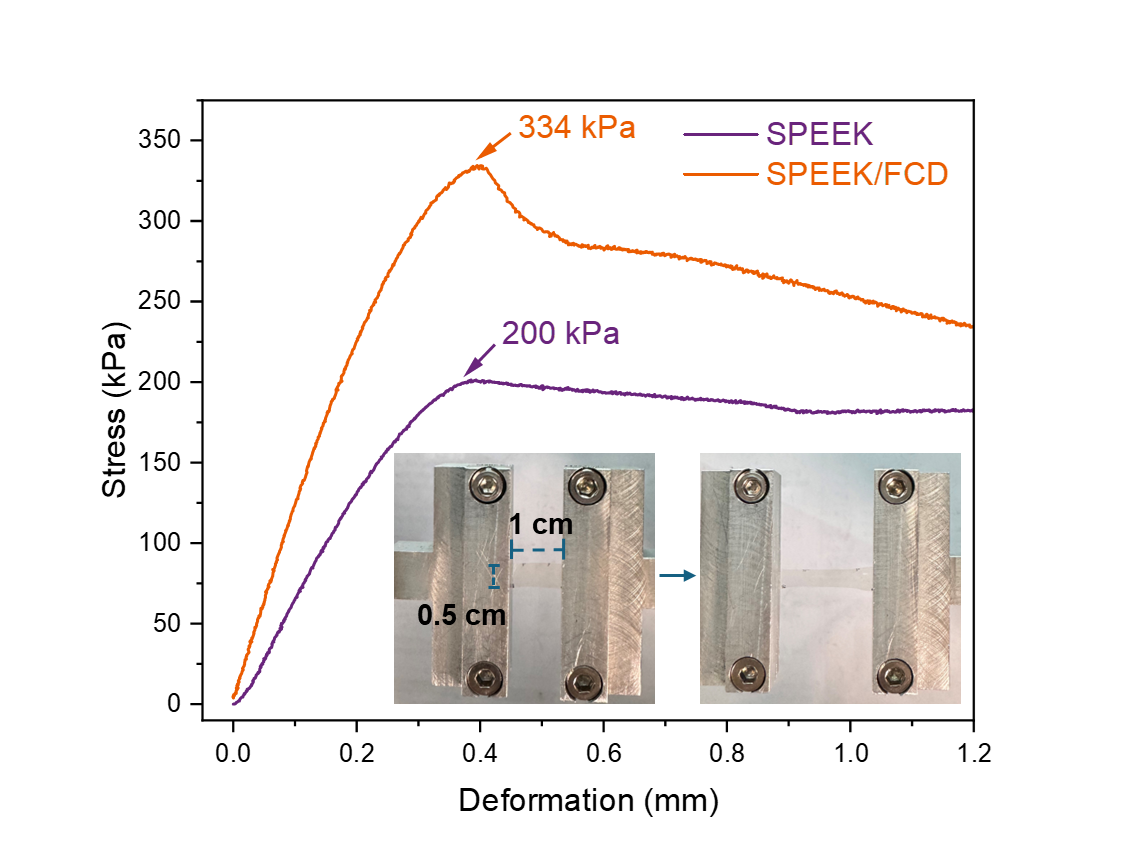


**Figure S15.** Tensile stress-strain curves of SPEEK and SPEEK/FCD films (50 µm in thickness).

**
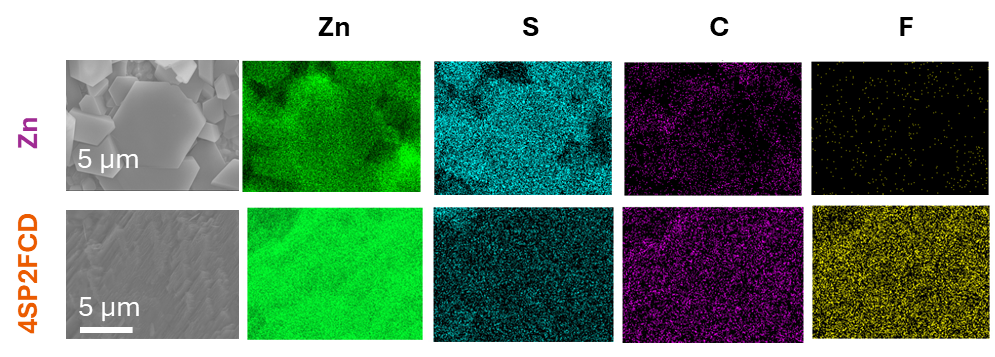
Figure S16.** SEM and corresponding EDS elemental mapping of Zn electrodes after 50 cycles for bare Zn and 4SP2FCD electrode.


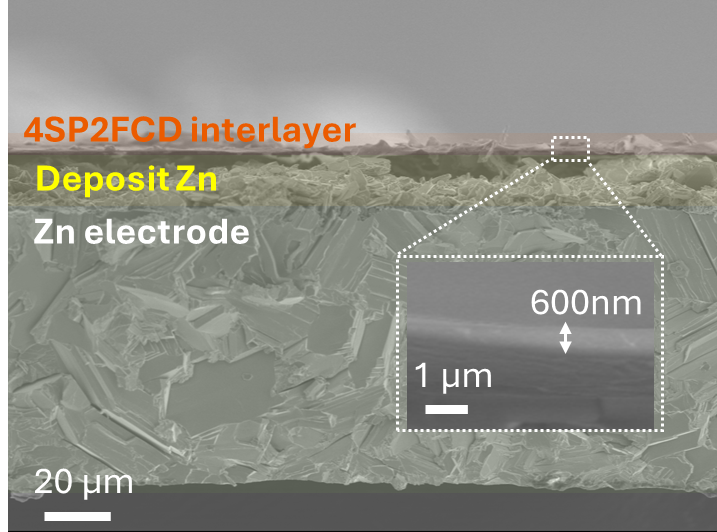


**Figure S17.** Cross-sectional SEM image of the 4SP2FCD electrode after 50 cycles. (inset: high resolution SEM image of the interlayer)


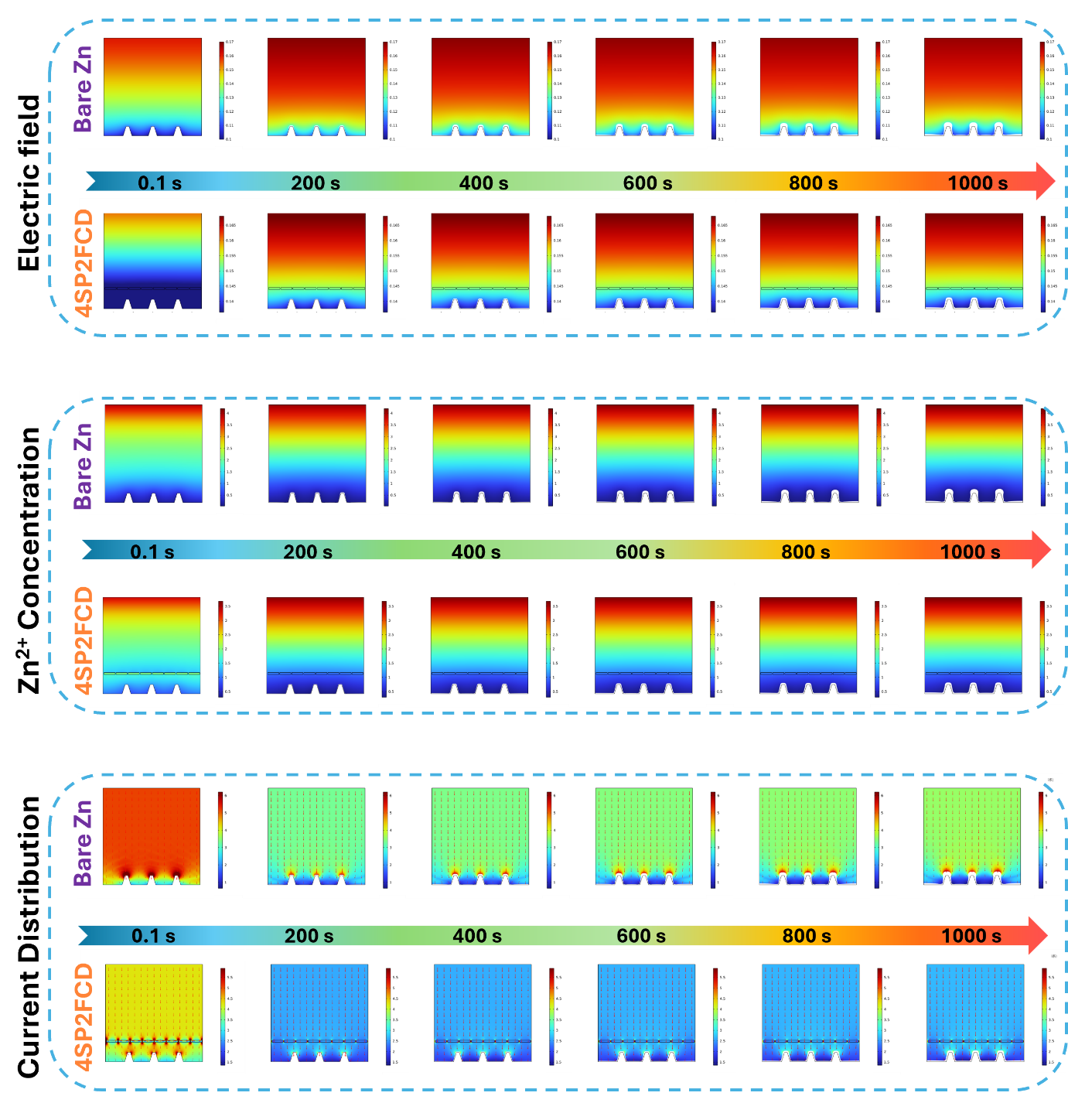


**Figure S18.** COMSOL simulation results comparing bare Zn and 4SP2FCD electrodes over a 0-1000 s deposition period.


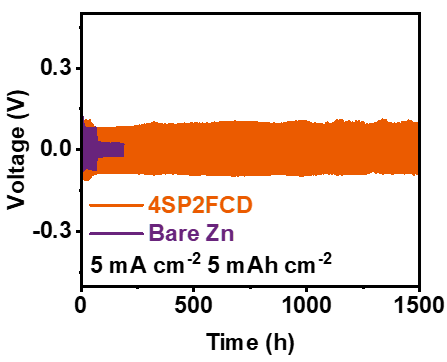


**Figure S19.** Long-term cycling performance of Zn||Zn symmetric cells using bare Zn and 4SP2FCD electrodes at 5 mA cm^−2^ and 5 mAh cm^−2^.


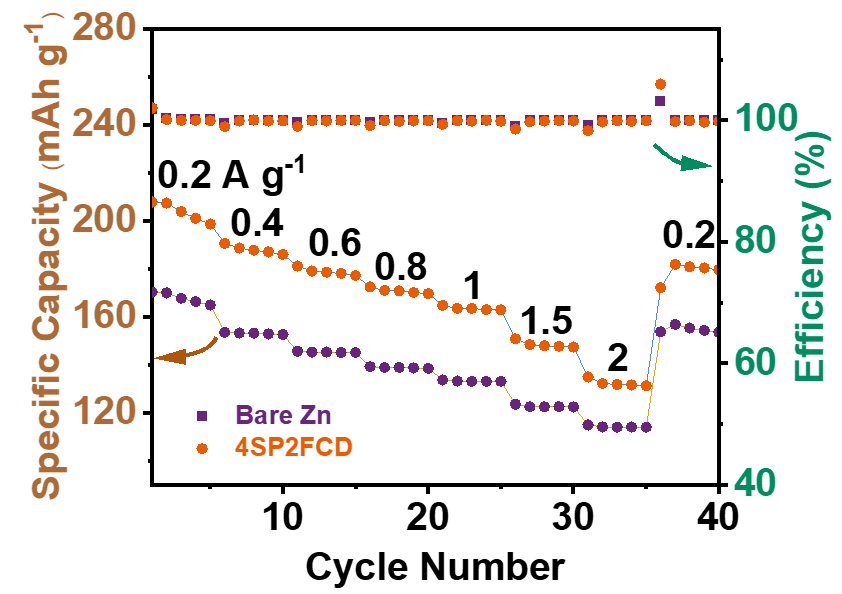


**Figure S20.** Rate performance and Coulombic Efficiency of Zn||V_2_O_5_ full cells using bare Zn and 4SP2FCD electrodes at various current densities from 0.2 to 2.0 A g^−1^_._

**Table S1. Comparison of Zn Anode Interlayers under Different CD/SC Conditions**

| Interlayer | Electrolyte | CD/SC  (mA cm^-2^/mAh cm^-2^) | Cycle life (h) | Thickness | Ref. |
| --- | --- | --- | --- | --- | --- |
| This Work | **2 M ZnSO_4_** | **1/1** | **5500** | **600 nm** | - |
|  |  | **5/5** | **1500** |  |  |
| NaTi_2_(PO_4_)_3_ | 2 M ZnSO_4_ | 1/1 | 260 | 20-25 µm | ^[6]^ |
| SPEEK | 1 M ZnSO_4_ | 5/10 | 350 | 5 µm | ^[7]^ |
| SPEEK | 1 M ZnSO_4_ | 1/1 | 475 | 1 µm | ^[8]^ |
| MOFs-SPEEK | 2 M ZnSO_4_ | 5/5 | 700 | 8.6 µm | ^[9]^ |
| PES-SPEEK | 2 M ZnSO_4_ | 5/5 | 800 | 10 µm | ^[10]^ |
| H-SPEEK | 2 M ZnSO_4_ | 2/2 | 1230 | 6.8 µm | ^[11]^ |
| SPEEK | 2 M ZnSO_4_ | 0.5/0.5 | 1600 | 7 µm | ^[12]^ |
| CDs | 2 M ZnSO_4_ | 1/1 | 3000 | 20 µm | ^[13]^ |
| F-CD | 2 M ZnSO_4_ | 1/1 | 3500 | 25 µm | ^[1]^ |
| DB-Cm | 2 M ZnSO_4_ | 1/1 | 1200 | 8.1 µm | ^[14]^ |

**Reference**

[1] Z. Ge, L. Xu, Y. Xu, J. Wu, Z. Geng, X. Xiao, W. Deng, G. Zou, H. Hou, X. Ji, "Multifunctional fluorinated carbon dots artificial interface layer coupled with in-situ generated Zn2+ conductor interlayer enable ultra-stable Zn anode," *Nano Energy*, 119, (2024): 109053. <https://doi.org/10.1016/j.nanoen.2023.109053>

[2] G. G. Kresse, J. J. Furthmüller, "Efficient Iterative Schemes for Ab Initio Total-Energy Calculations Using a Plane-Wave Basis Set," *Physical review. B, Condensed matter*, 54, (1996): 11169.

[3] G. Kresse, D. Joubert, "From ultrasoft pseudopotentials to the projector augmented-wave method," *Physical Review B*, 59, 3 (1999): 1758.

[4] J. P. Perdew, K. Burke, M. Ernzerhof, "Generalized Gradient Approximation Made Simple," *Phys. Rev. Lett.*, 77, 18 (1996): 3865.

[5] P., E., Blöchl, "Projector augmented-wave method," *Physical Review B*, 50, (1994): 17953.

[6] M. Liu, J. Cai, H. Ao, Z. Hou, Y. Zhu, Y. Qian, "NaTi2 (PO4) 3 solid‐state electrolyte protection layer on Zn metal anode for superior long‐life aqueous zinc‐ion batteries," *Advanced Functional Materials*, 30, 50 (2020): 2004885.

[7] H. Fan, M. Wang, Y. Yin, Q. Liu, B. Tang, G. Sun, E. Wang, X. Li, "Tailoring interfacial Zn2+ coordination via a robust cation conductive film enables high performance zinc metal battery," *Energy Storage Materials*, 49, (2022): 380. <https://doi.org/10.1016/j.ensm.2022.04.031>

[8] Q. Jian, Y. Wan, Y. Lin, M. Ni, M. Wu, T. Zhao, "A highly reversible zinc anode for rechargeable aqueous batteries," *ACS Applied Materials & Interfaces*, 13, 44 (2021): 52659.

[9] H. Fan, M. Li, E. Wang, "Anion-functionalized interfacial layer for stable Zn metal anodes," *Nano Energy*, 103, (2022): 107751.

[10] W. Dong, C. Liu, X. Ji, H. Yao, J. Li, H. Du, S. Cheng, "Construction of Cation-Sieving Function Layers Enabling Dendrite-Free Zinc Metal Anodes for Durable Aqueous Systems," *Small Methods*, 8, 6 (2024): 2300799. <https://doi.org/10.1002/smtd.202300799>

[11] J. Wang, Y. Yu, R. Chen, H. Yang, W. Zhang, Y. Miao, T. Liu, J. Huang, G. He, "Induced Anionic Functional Group Orientation-Assisted Stable Electrode-Electrolyte Interphases for Highly Reversible Zinc Anodes," *Advanced Science*, 11, 25 (2024): 2402821. <https://doi.org/10.1002/advs.202402821>

[12] Y. Wang, Y. Wang, C. Chen, X. Chen, Q. Zhao, L. Yang, L. Yao, R. Qin, H. Wu, Z. Jiang, "Optimizing the sulfonic groups of a polymer to coat the zinc anode for dendrite suppression," *Chemical Communications*, 57, 43 (2021): 5326.

[13] H. Zhang, S. Li, L. Xu, R. Momen, W. Deng, J. Hu, G. Zou, H. Hou, X. Ji, "High-Yield Carbon Dots Interlayer for Ultra-Stable Zinc Batteries," *Advanced Energy Materials*, 12, 26 (2022): 2200665. <https://doi.org/10.1002/aenm.202200665>

[14] Z. Jiang, Z. Du, R. Pan, F. Cui, G. Zhang, S. Lei, G. He, K. Yin, L. Sun, "Electrosynthesis of Metal–Organic Framework Interlayer to Realize Highly Stable and Kinetics-Enhanced Zn Metal Anode," *Advanced Energy Materials*, 14, 44 (2024): 2402150. <https://doi.org/10.1002/aenm.202402150>
